# The kallikrein-kinin system modulates CD4^+^ T cell function and contributes to T cell migration into the brain after stroke

**DOI:** 10.1186/s12974-026-04034-4

**Published:** 2026-09-05

**Authors:** Nadine Gausmann, Steffen Haupeltshofer, Rebecca D. Szepanowski, Sina Luppus, Egor Dzyubenko, Jasmin Bahr, Stine Mencl, Janine Gronewold, Dirk M. Hermann, Michael Forsting, Cornelius Deuschl, Jan Buer, Astrid M. Westendorf, Christoph Kleinschnitz, Ana I. Casas, Wiebke Hansen

**Affiliations:** 1 Institute of Medical Microbiology, University Hospital Essen, Hufelandstraße 55, 45147 Essen, Germany; 2 Department of Neurology and Center for Translational Neuro- and Behavioral Sciences (C-TNBS), Clinic for Neurology, University Hospital Essen, Essen, Germany; 3 Translational Neurology Group, Department of Clinical Science, Lund University, Lund, Sweden; 4 Institute of Diagnostic and Interventional Radiology and Neuroradiology, University Hospital Essen, Essen, Germany; 5 Department of Pharmacology and Personalised Medicine, Faculty of Health, Medicine and Life Sciences, Maastricht University, Maastricht, The Netherlands

**Keywords:** Ischemic stroke, Thromboinflammation, Kallikrein-kinin system, Plasma kallikrein, T cells, Migration

## Abstract

Ischemic stroke elicits a sustained thromboinflammatory response that critically shapes secondary brain injury. The kallikrein-kinin system (KKS) has been identified as the interface of vascular injury and inflammatory processes; however, its role in adaptive immunity remains poorly defined. Here, we show that the KKS is associated with pathogenic CD4^+^ T cell activation and trafficking. In both ischemic stroke patients and mice subjected to transient middle cerebral artery occlusion (tMCAO), circulating CD4^+^ T cells exhibited a conserved, activated, and trafficking-competent phenotype characterized by upregulation of adhesion/migration markers, indicating a translationally preserved systemic adaptive immune response. Functionally, serum from stroke mice subacutely treated with a plasma kallikrein (PK)-neutralizing antibody (ɑPK) attenuated CD4^+^ T cell activation, proliferation, expression of adhesion/migration markers, and pro-inflammatory cytokine production in vitro. PK selectively enhanced CD4^+^ T cell adhesion and migration, while its downstream metabolite Des-Arg^9^-bradykinin (Des-Arg^9^-BK) induced a pronounced pro-inflammatory, pro-migratory phenotype and potentiated chemokine-driven transendothelial migration in vitro. Strikingly, delayed PK inhibition in vivo was associated with reduced T cell accumulation in the ischemic brain and increased circulating CD4^+^ T cell frequencies, suggesting impaired central nervous system (CNS) infiltration dynamics. Together, these findings support a role for KKS signaling in shaping neuroimmune interactions after ischemic stroke by enhancing endothelial adhesiveness and facilitating CD4^+^ T cell migration to the ischemic tissue. By linking thromboinflammation and adaptive immunity, the KKS emerges as a promising therapeutic target to selectively modulate neuroimmune interactions and potentially improve functional recovery after ischemic stroke.

**Supplementary Information:**

The online version contains supplementary material available at https://doi.org/10.1186/s12974-026-04034-4.

## Background

Ischemic stroke poses a major and growing public-health challenge. Globally, the burden of stroke continues to increase with millions of new cases reported annually, where the vast majority are ischemic in origin [1]. Current standard-of-care therapies, i.e., intravenous thrombolysis with recombinant tissue-plasminogen activator or endovascular mechanical thrombectomy focus on rapid reperfusion and restoration of flow. However, these interventions do not directly tackle the complex thromboinflammatory environment following the ischemic event [2]. Consequently, while reperfusion initially limits infarct expansion, secondary injury and functional recovery remain challenging, highlighting the urgent need for a deeper mechanistic understanding of the multifaceted stroke pathobiology.

The immune system has emerged as a key player in stroke recovery [3]. Following ischemia, brain cells release damage-associated molecular patterns (DAMPs) into the circulation and perivascular spaces. DAMPs trigger the activation of the endothelium, thereby promoting leukocyte infiltration, tissue edema, blood-brain barrier (BBB) disruption, and progressive brain injury [4, 5]. Among infiltrating cells, T cells are increasingly recognized as critical modulators of both neuronal damage and repair [3]. T cells can be further subdivided into CD4^+^ helper T cells (Th), CD8^+^ cytotoxic T cells, regulatory T cells, and natural killer (NK) T cells according to the differential expression of surface markers [6]. Importantly, CD4^+^ T cells tend to drive immunomodulatory and pro-inflammatory responses, activating other inflammatory cells [3], confirmed in T cell-depleted mice showing a clear protective phenotype after stroke [7, 8]. In ischemic stroke, peripheral T cells become activated and start a well-defined cascade of vascular interactions, i.e., tethering, rolling, arrest, and adhesion to the endothelium, and finally transmigration across the cerebrovascular endothelium. Once within the brain parenchyma or the perivascular niche, T cells can exert pro-inflammatory effects through cytokine production and glial cell activation or, depending on the subtype, timing, and microenvironmental cues, contributing to recovery processes [9]. T cell trafficking is mediated through the sequential interplay of adhesion and/or signaling molecules including integrins on immune cells and their respective receptors on the activated endothelium [10]. Following stroke, endothelial cells rapidly acquire a pro-inflammatory phenotype, expressing different adhesion molecules that interact with T cell integrins, promoting T cell infiltration into the central nervous system (CNS). In detail, P-selectin expression on the endothelial surface enables initial leukocyte tethering via CD44 post-stroke, thereby establishing transient contacts required for rolling [11]. Rolling, arrest, and firm adhesion of lymphocytes are mediated by direct binding of specific integrins to their endothelial ligands, many of which are upregulated after brain ischemia [12, 13]. Prominent integrin-ligand pairs include LFA-1 on T cells interacting with ICAM-1, and VLA-4 binding to endothelial VCAM-1, both playing central roles in T cell recruitment to the inflamed CNS [12]. Chemokine gradients further refine the T cell infiltration process. CXCL10, a well-characterized chemoattractant secreted by astrocytes, endothelial cells, and neurons in response to stimuli, binds to CXCR3, highly expressed by pro-inflammatory type 1 helper T cells (Th1) and cytotoxic CD8^+^ T cells [13]. CXCL12, originally named stromal cell-derived factor-1β (SDF-1β) and ligand of CXCR4, is also expressed by multiple CNS cell types and strongly induced after ischemia. It provides an additional chemotactic axis that shapes the phenotype of T cell accumulation in the injured brain [14].

In transient rodent stroke models, T cell infiltration into the brain parenchyma rises markedly from day 1 to day 7 after reperfusion [15, 16], although accumulation can continue up to 30 days [12]. In humans, T cell numbers increase as early as day 1 after stroke onset and can persist for weeks to months [17]. Yet, despite this broad temporal pattern, the infiltration dynamics of distinct T cell populations in patients remain largely undefined [12]. Emerging evidence indicates that T cell-vascular interactions are key drivers of thromboinflammatory processes, promoting platelet activation, thrombus formation, microvascular dysfunction, and ultimately delayed cerebral injury [2]. Nonetheless, definitive mechanistic evidence regarding the direct contribution of T cells to coagulation and vascular response post-stroke is still limited, highlighting a critical gap around the post-stroke immune-vascular crosstalk. In this line, the contact plasma kallikrein-kinin system (KKS) has emerged as a key interface between coagulation, inflammation, and vascular pathology in ischemic stroke. It consists of serially connected serine proteases (plasma kallikrein, PK; factor XII, FXII; high-molecular-weight kininogen, HMWK) and downstream mediators (bradykinin, BK; Des-Arg^9^-bradykinin, Des-Arg^9^-BK) regulating thrombus formation, vascular permeability, and BBB integrity [18]. Experimental inhibition of PK in stroke models has been shown to reduce infarct size while improving neuromotor function, reducing microthrombosis, and enhancing BBB stability. Indeed, pharmacological blockade of the KKS decreases immune cell infiltration [5], although the specific impact on T cell recruitment remains to be fully characterized (Suppl. Figure 1).

Here, we investigated the direct effect of PK inhibition on T cell response and function to unravel the complex interface between vascular impairment and inflammatory response in the subacute phase after ischemic stroke. By targeting this vascular-immune bridge, we propose a mechanistic strategy that may augment reperfusion-based approaches and support functional recovery.

## Results

### Enhanced activation and trafficking-associated phenotype of CD4^+^ T cells following ischemic stroke

**Fig. 1:**
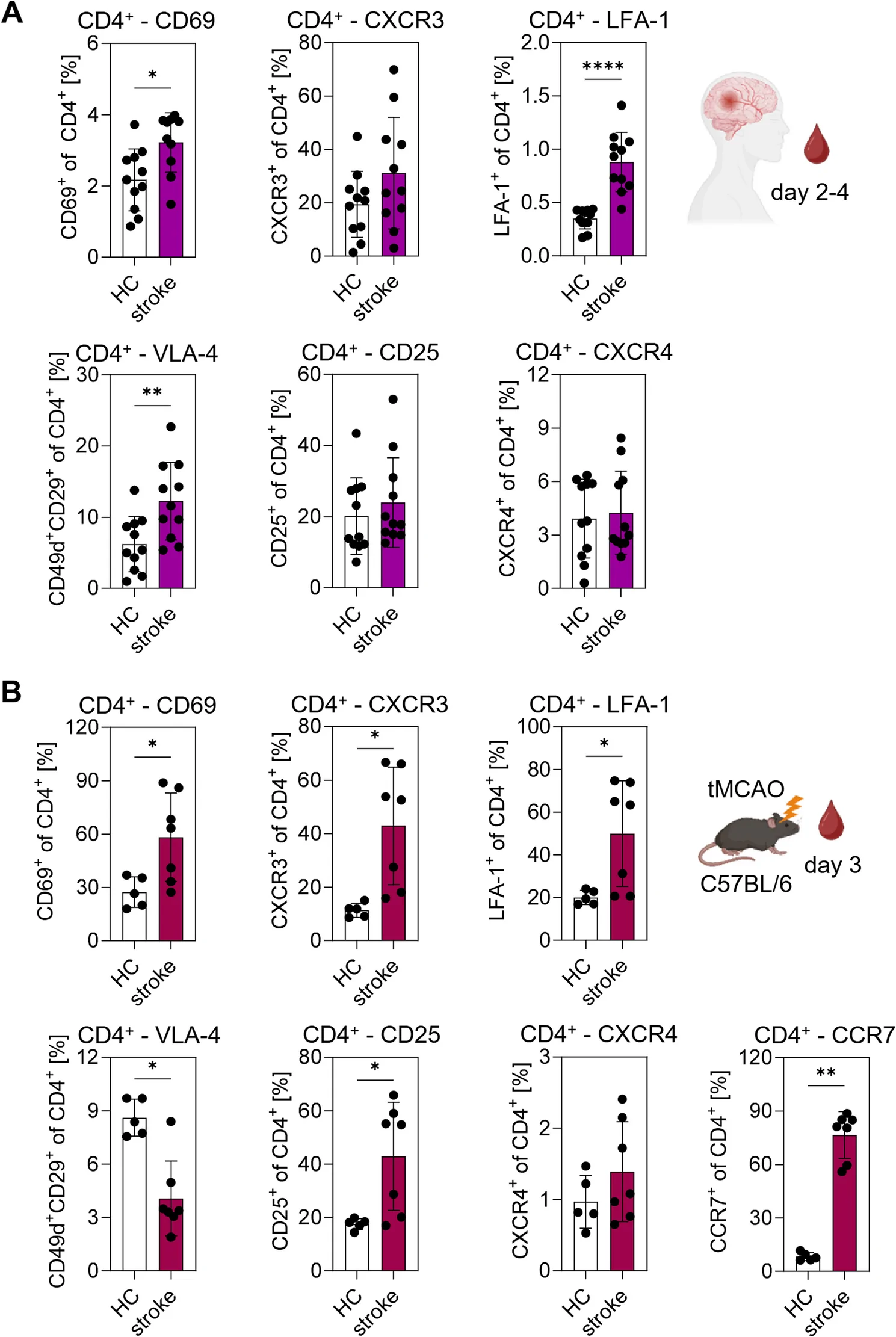
Stroke increases CD4^+^ T cell activation and expression of adhesion/migration-associated molecules in blood of human patients and of tMCAO mice. **A** Peripheral blood was collected from healthy controls (HC) and ischemic stroke patients 2-4 days after onset. Peripheral blood mononuclear cells (PBMCs) were isolated and CD4^+^ T cells were analyzed by flow cytometry for CD69, CXCR3, LFA-1, VLA-4, CD25, and CXCR4 expression (mean ± SD, *n* = 11). **B** C57BL/6 mice were subjected to 60-min transient middle cerebral artery occlusion (tMCAO) or left untreated (HC). Murine CD4^+^ T cells were isolated from the blood on day 3 and analyzed for activation and adhesion/migration-associated markers by flow cytometry. Data represent two independent experiments (mean ± SD, *n* = 5-7). Statistical significance was determined by unpaired Student’s t-test or Mann-Whitney test, * *p* < 0.05, ** *p* < 0.01, **** *p* < 0.0001. Created with BioRender.com

To evaluate the translational relevance of the T cell response observed in preclinical models post-stroke [19–21], we analyzed the activation and adhesion/migration profile of circulating T cells in stroke patients (Suppl. Table 1). Peripheral blood samples were collected 2–4 days after stroke onset, followed by isolation and phenotypic characterization of CD4^+^ T cells by flow cytometry (Suppl. Figure 2 A). Compared to healthy controls, stroke patients exhibited significantly increased relative numbers of CD4^+^ T cells expressing the early activation marker CD69 and the integrins LFA-1 and VLA-4, indicating an enhanced trafficking-competent phenotype. CXCR3 and CD25 expressing CD4^+^ T cells showed a non-significant trend towards elevation, whereas CXCR4 expression remained unchanged (Fig. 1A). Importantly, increased expression of the activation marker CD69, in addition to the adhesion-associated molecule LFA-1 persisted at 5–7 days after stroke accompanied by elevated circulating CD4^+^ T cell frequencies (Suppl. Figure 2B-C), altogether suggesting a sustained systemic immune response during the subacute phase. Similar to patients, mice subjected to transient middle cerebral artery occlusion (tMCAO) showed an increased relative number of CD4⁺ T cells expressing activation, migration and adhesion markers, including CD69, CXCR3, LFA-1, CD25, and CCR7 at 3 days post-stroke in peripheral blood (Fig. 1B, Suppl. Figure 3). The close alignment between patient and preclinical data highlights the translational validity of the model, showing conserved post-stroke T cell activation and trafficking signatures across species.

### Serum from αPK-treated tMCAO mice modulates CD4^+^ T cell responses in vitro

Despite extensive evidence for T cell-vascular interactions in post-stroke thromboinflammation [3], the direct contribution of coagulation and vascular responses to T cells remains unclear. Importantly, the KKS represents a key molecular player promoting thrombosis and inflammation after stroke, mediating vascular permeability and BBB disruption [18]. Indeed, pharmacological inhibition of the KKS during the subacute stroke phase using a PK-neutralizing antibody (αPK) significantly improved the vascular response post-stroke associated with reduced CD3^+^ T cell infiltration into the ischemic brain [5, 22].

**Fig. 2:**
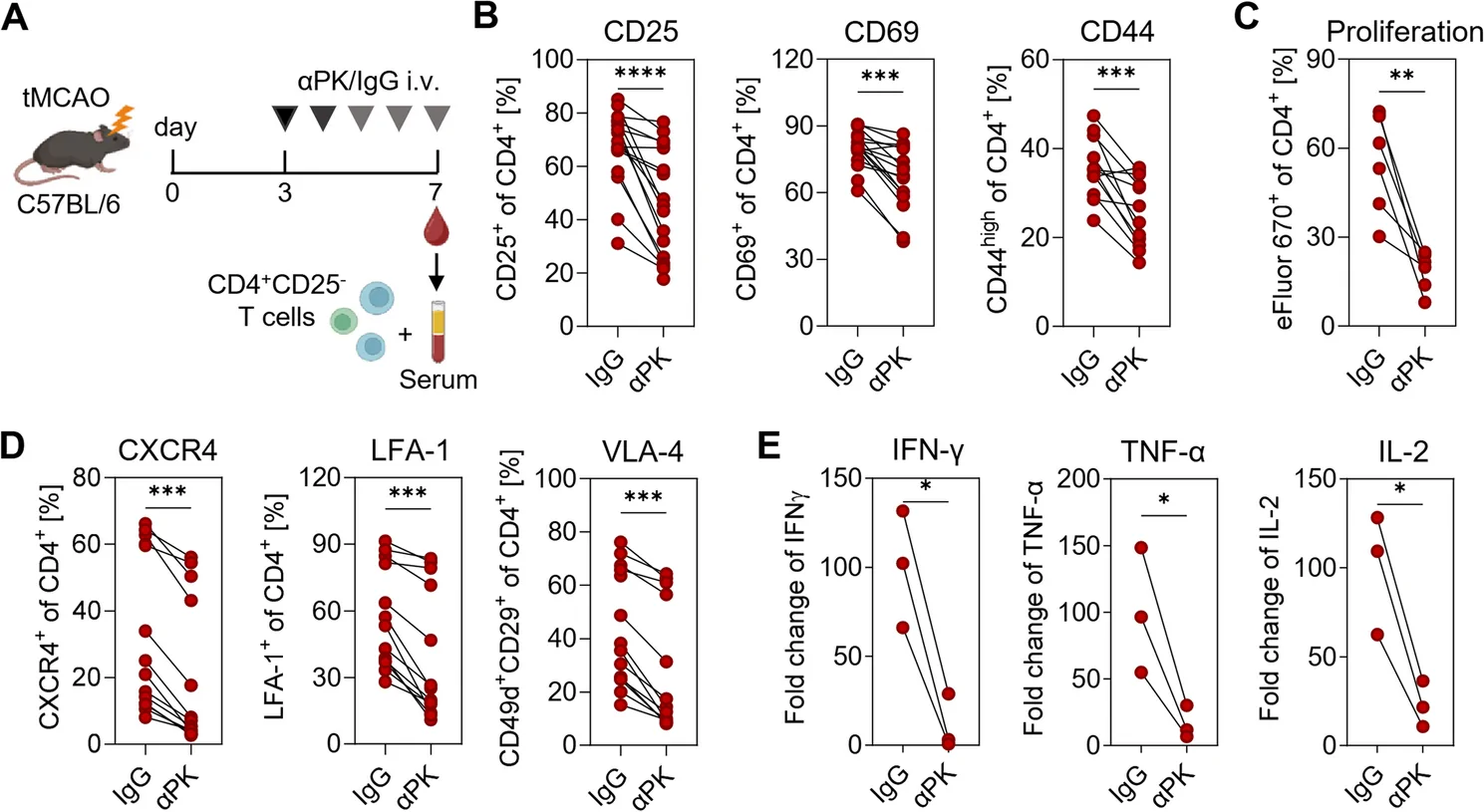
Serum from plasma kallikrein-neutralizing antibody-treated tMCAO mice interferes with CD4^+^ T cell responses in vitro. **A** C57BL/6 mice were subjected to 60-min transient middle cerebral artery occlusion (tMCAO). Mice were treated with isotype immunoglobulin G (IgG) or a plasma kallikrein-neutralizing antibody (αPK) at days 3 and 4 (10 µg) and days 5 to 7 (5 µg). Serum was collected on day 7 and added to αCD3/αCD28-stimulated splenic CD4^+^CD25^-^ T cells from naïve C57BL/6 mice. **B** CD4^+^ T cells were analyzed by flow cytometry for expression of activation markers (24 h), **C** proliferation (72 h), and **D** expression of adhesion/migration-associated molecules (48 h). Data represent two to three independent experiments (mean ± SD, *n* = 6-16). **E** Cytokine concentrations in cell culture supernatants were quantified by Luminex after 72 h. Data represent three independent biological replicates of serum probes tested in one experiment (mean ± SD, *n* = 3). Statistical significance was determined by paired Student’s t-test or Wilcoxon test, * *p* < 0.05, ** *p* < 0.01, *** *p* < 0.001, **** *p* < 0.0001. Created with BioRender.com

To examine the direct impact of KKS modulation on the CD4^+^ T cell response, splenic CD4⁺CD25⁻ T cells from naïve C57BL/6 mice were stimulated with αCD3/αCD28, and exposed to serum collected 7 days after tMCAO from αPK- or immunoglobulin-G (IgG)-treated mice during the subacute stroke phase (Fig. 2A). T cell activation, proliferative capacity, adhesion/migration marker expression, and cytokine production were subsequently analyzed. Exposure of splenic CD4⁺CD25⁻ T cells to serum from αPK-treated stroke mice resulted in reduced frequencies of CD4^+^ T cells expressing the activation-associated markers CD25, CD69, and CD44 (Fig. 2B), alongside decreased CD4^+^ T cell proliferation (Fig. 2C). Additionally, serum from αPK-treated stroke mice markedly downregulated the frequencies of CD4^+^ T cells expressing the adhesion and migration molecules CXCR4, LFA-1, and VLA-4 after stimulation (Fig. 2D). Luminex analysis of T cell culture supernatants further suggests that the presence of serum from αPK-treated stroke mice reduces the secretion of key pro-inflammatory cytokines including IFN-γ, TNF-α, and IL-2 in stimulated CD4^+^ T cells (Fig. 2E). To exclude differences in total protein concentration as a confounding factor, serum protein levels were measured by Bicinchoninic acid assay (BCA) at day 7 post-stroke showing comparable levels between IgG- and αPK-treated mice (Suppl. Figure 4). Collectively, these data demonstrate that pharmacological inhibition of PK reshapes the systemic post-stroke environment resulting in attenuated activation, reduced trafficking-associated phenotype, and diminished pro-inflammatory effector function of CD4⁺ T cells in vitro.

### KKS components directly modulate CD4^+^ T cell function and phenotype

**Fig. 3:**
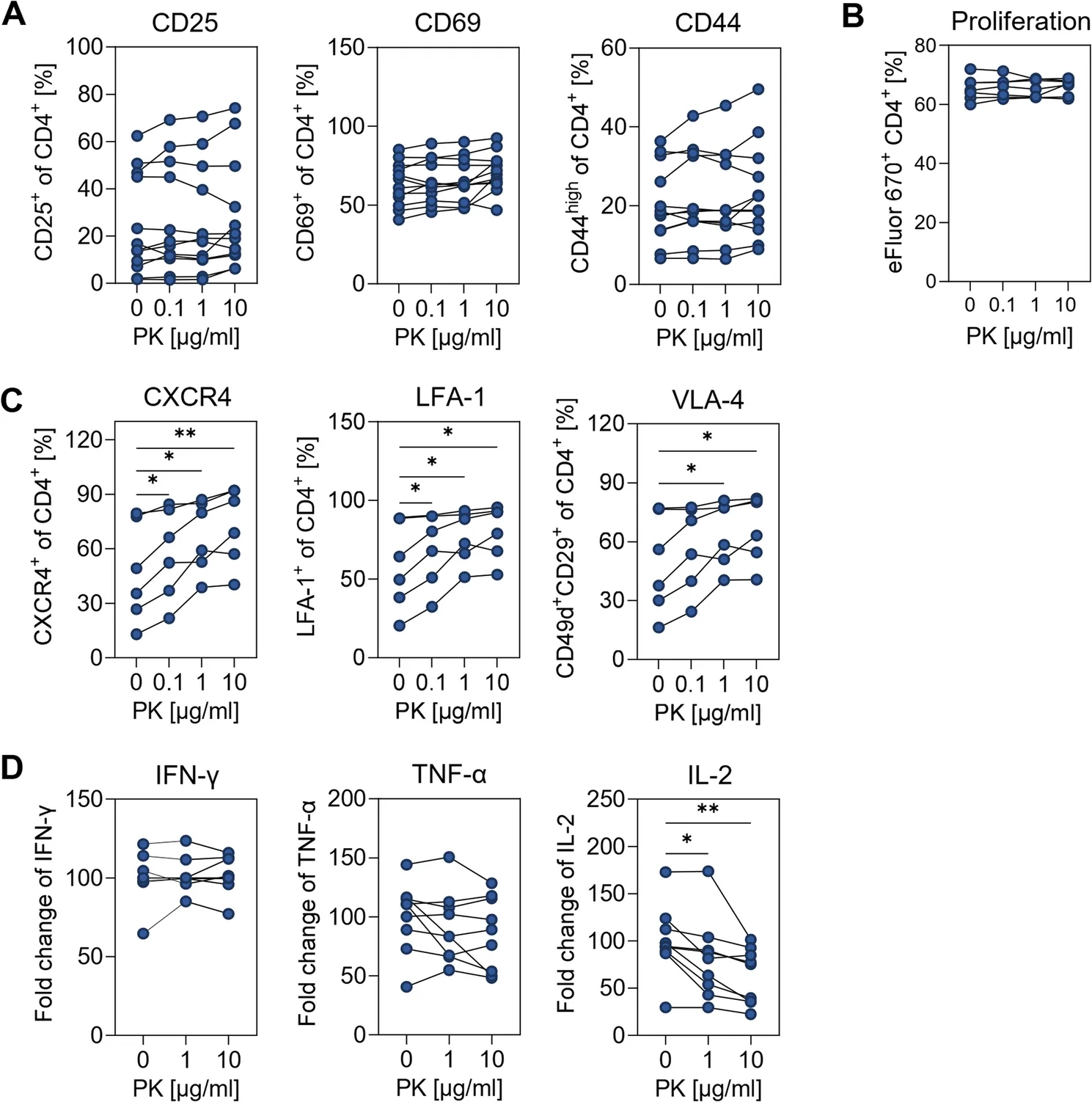
Plasma kallikrein enhances adhesion/migration-associated molecule expression of CD4^+^ T cells in vitro. Splenic CD4^+^CD25^-^ T cells from naïve C57BL/6 mice were stimulated with αCD3/αCD28 in the presence of 0, 0.1, 1 or 10 µg/ml plasma kallikrein (PK). **A** CD4^+^ T cells were analyzed by flow cytometry for expression of activation markers (24 h), **B** proliferation (72 h), and **C** expression of adhesion/migration-associated molecules (48 h). Data represent two to four independent experiments (mean ± SD, *n* = 6-12). **D** Cytokine concentrations in cell culture supernatants were quantified by Luminex after 72 h. Data represent three independent experiments (mean ± SD, *n* = 9). Statistical significance was determined by RM one-way ANOVA, * *p* < 0.05, ** *p* < 0.01

PK is a critical direct regulator of BBB integrity through its proteolytic activity, degrading tight junction proteins and thereby facilitating leukocyte trafficking to the brain parenchyma [23]. In line with this established vascular role, our data further suggest that pharmacological PK inhibition modifies the post-stroke systemic milieu, attenuating the pro-inflammatory T cell phenotype upon stroke. Since delayed PK inhibition in tMCAO mice resulted in an about two-fold decrease in PK and BK serum concentrations 5 and 7 days post-treatment [5], we assessed whether these KKS components exert a direct effect on T cells independent of vascular intermediates. Therefore, splenic CD4^+^CD25^−^ T cells isolated from naïve C57BL/6 mice were stimulated with αCD3/αCD28 and exposed to increasing concentrations of PK in vitro. PK exposure did not significantly affect CD4^+^ T cell activation and proliferative capacity (Fig. 3A-B). In contrast, PK induced a marked, concentration-dependent elevation of adhesion and migration markers expressed on CD4^+^ T cells (Fig. 3C). The IL-2 concentration in culture supernatants was reduced in a concentration-dependent manner, despite IFN-γ and TNF-α release not being affected by the treatment (Fig. 3D). Together, these findings indicate that PK selectively modulates CD4^+^ T cell adhesion properties, enhancing expression of migratory markers without inducing a uniform pro-inflammatory phenotype.

**Fig. 4:**
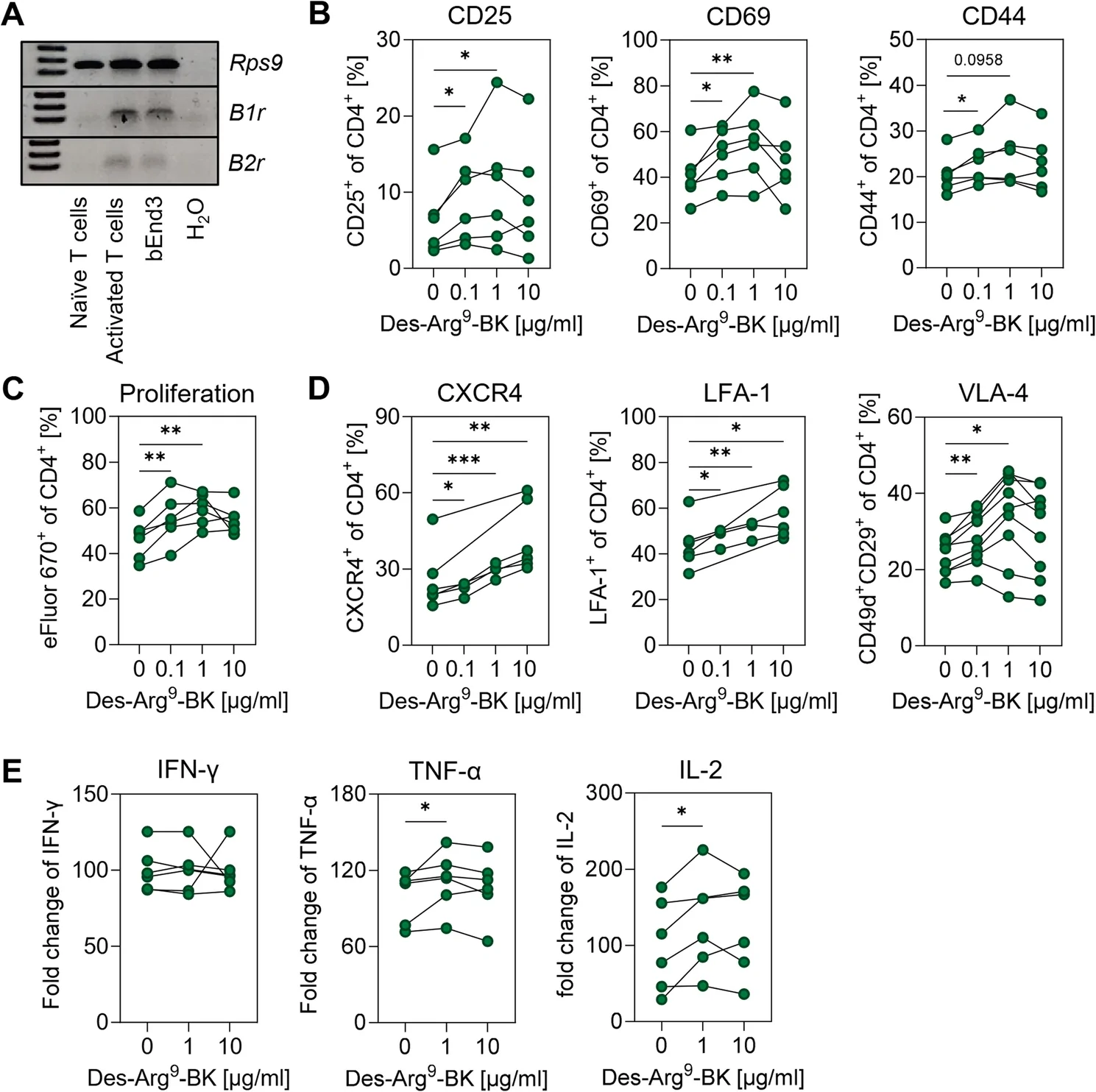
Des-Arg^9^-bradykinin enhances pro-inflammatory CD4^+^ T cell responses and adhesion/migration-associated molecule expression in vitro. **A** Splenic CD4^+^CD25^-^ T cells from naïve C57BL/6 mice were stimulated with αCD3/αCD28 or left unstimulated for 48 h. RNA was isolated, reverse-transcribed to cDNA, and analyzed by PCR for expression of kinin receptors B1 (B1r) and B2 (B2r). Endothelial cells (bEnd3) served as positive control, and ribosomal protein S9 (Rps9) as housekeeping gene. **B**-**E** Splenic CD4^+^CD25^-^ T cells from naïve C57BL/6 mice were stimulated with αCD3/αCD28 in the presence of 0, 0.1, 1 and 10 µg/ml Des-Arg^9^-bradykinin (Des-Arg^9^-BK). **B** CD4^+^ T cells were analyzed by flow cytometry for expression of activation markers (24 h), **C** proliferation (72 h), and **D** expression of adhesion/migration-associated molecules (48 h). Data represent two to three independent experiments (mean ± SD, *n* = 6-9). **E** Cytokine concentrations in cell culture supernatants were quantified by Luminex after 72 h. Data represent two independent experiments (mean ± SD, *n* = 6). Statistical significance was determined by RM one-way ANOVA, Friedman test or Mixed-effects analysis, * *p* < 0.05, ** *p* < 0.01, *** *p* < 0.001

Given that αPK treatment also reduces circulating BK levels [5], we hypothesized a potential direct role of BK promoting T cell responses relevant to post-stroke neuroinflammation. To validate this, we first confirmed the expression of the kinin receptors B1 (B1R) and B2 (B2R) on αCD3/αCD28-activated CD4^+^ T cells, indicating their capacity to directly sense BK/Des-Arg^9^-BK signaling (Fig. 4A). Consistent with our findings, B1R and B2R protein expression has previously been reported in human T cell populations, including peripheral T cells from patients with multiple sclerosis, and leukocyte subsets from healthy individuals [24–26]. Exposure of stimulated CD4^+^CD25^−^ T cells to Des-Arg^9^-BK, the metabolically active form of BK [27], induced a robust pro-inflammatory phenotype characterized by enhanced activation, proliferation (Fig. 4B-C), upregulation of adhesion/migration molecules (Fig. 4D), and elevated secretion of pro-inflammatory cytokines (Fig. 4E). To exclude potential sex-dependent differences, CD4^+^ T cells were isolated from female and male mice and analyzed separately, revealing no detectable differences in the upregulation of migration- and adhesion-associated molecules upon PK or Des-Arg^9^-BK treatment (Suppl. Figure 5 A-B). In contrast to the partial effects observed after PK treatment, its downstream mediator Des-Arg^9^-BK exerts more potent pro-inflammatory effects, suggesting that therapeutic targeting of upstream PK mitigates post-stroke neuroinflammation by limiting Des-Arg^9^-BK-mediated immune activation.

### Des-Arg^9^-BK enhances CD4^+^ T cell adhesion and migration capacity

**Fig. 5:**
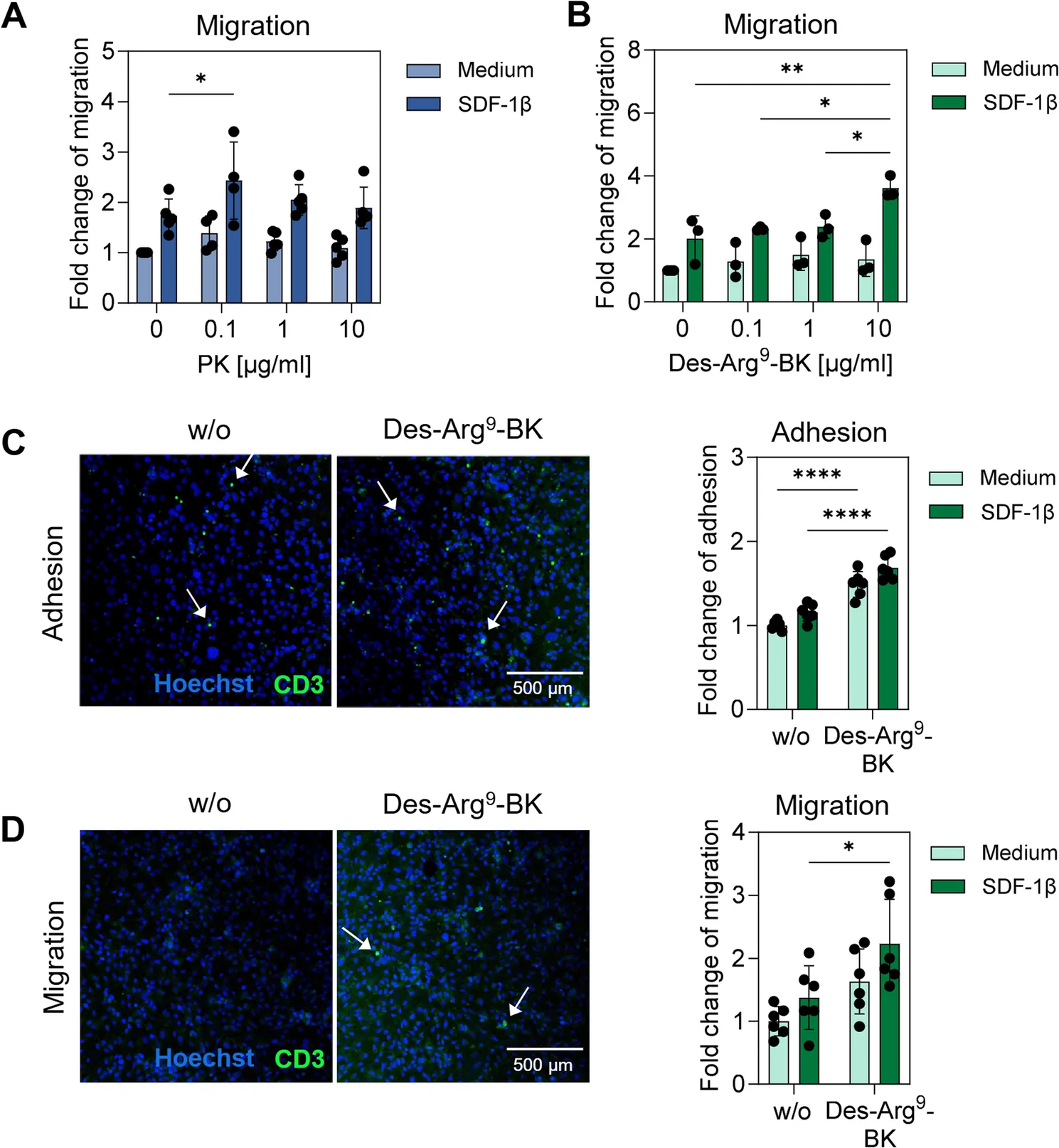
Des-Arg^9^-bradykinin promotes adhesion and migration of CD4^+^ T cells in vitro. **A**-**B** Splenic CD4^+^CD25^-^ T cells from naïve C57BL/6 mice were stimulated with αCD3/αCD28 in the presence of 0, 0.1, 1 or 10 µg/ml plasma kallikrein (PK) or Des-Arg^9^-bradykinin (Des-Arg^9^-BK) for 48 h. Subsequently, 0.6 x 10^6^ cells/well were transferred to transwell inserts (5 µm pore size), coated with fibronectin. After 4 h, all cells that have migrated across the transwell membrane into the lower chamber were collected and counted to assess undirected (medium) and SDF-1β-directed migration. Data are expressed as fold change relative to undirected migration of untreated control cells (0 µg/ml PK/Des-Arg^9^-BK). Results represent three to five independent experiments (mean ± SD, *n* = 3-5). **C**-**D** Splenic CD4^+^CD25^-^ T cells from naïve C57BL/6 mice were stimulated with αCD3/αCD28 in the absence (w/o) or presence of 10 µg/ml Des-Arg^9^-BK for 48 h. Then, 0.5 x 10^6^ cells/well were transferred into transwell inserts (8 µm pore size) with membranes pre-coated with endothelial cells (bEnd3; 0.5 x 10^5^ cells/well). Adhesion and migration were assessed under undirected (medium) and SDF-1β-directed conditions. After 4 h, transwells were fixed with 4 % PFA and all cells without contact to the endothelium were washed away. T cells that adhered on top of the endothelium and T cells that transmigrated and still were in contact to the lower side of the endothelium were quantified by fluorescence microscopy (nuclei: blue; CD3^+^ T cells: green). One representative set of images is shown. Data are expressed as fold change relative to undirected adhesion/migration of control cells (w/o). Results represent six independent experiments (mean ± SD, *n* = 6). Statistical significance was determined by two-way ANOVA followed by (**A**-**B**) Sidák’s multiple comparisons test, or (**C**-**D**) Tukey’s multiple comparisons test, * *p* < 0.05, ** *p* < 0.01, **** *p* < 0.0001

Given the upregulation of adhesion- and migration-associated markers on CD4^+^ T cells following PK and Des-Arg^9^-BK exposure, we next evaluated whether these phenotypic changes translated into enhanced migratory capacity. Splenic CD4^+^CD25^−^ T cells isolated from naïve C57BL/6 mice were pre-incubated with αCD3/αCD28 and pre-treated with increasing concentrations of PK or Des-Arg^9^-BK for 48 h. Both undirected (basal) and chemokine-directed migration were subsequently evaluated using an in vitro transwell assay. In detail, directed migration was mediated by SDF-1β (CXCL12), the canonical ligand for CXCR4 [28], whose expression was upregulated by both PK and Des-Arg^9^-BK (Figs. 3C and 4D). PK pre-treatment significantly increased SDF-1β-directed migration at 0.1 µg/ml (Fig. 5A), whereas Des-Arg^9^-BK exerted a marked effect at 10 µg/ml (Fig. 5B). Towards more translational conditions, we employed a murine BBB transwell in vitro model using mouse brain microvascular endothelial cells (MBMECs, bEnd3). Des-Arg^9^-BK-pre-treated CD4^+^ T cells exhibited increased SDF-1β-induced adhesion to the endothelial monolayer compared to only αCD3/αCD28-activated controls (Fig. 5C). Similarly, Des-Arg^9^-BK pre-treatment also promoted directed transendothelial migration of CD4^+^ T cells across the MBMECs monolayer (Fig. 5D). To exclude an effect of Des-Arg^9^-BK-pre-treated CD4^+^ T cells on the endothelial barrier integrity, we measured transendothelial electrical resistance (TEER) using the transwell BBB model. TEER values were comparable between bEnd3 monocultures, bEnd3 cells co-cultured with untreated T cells, and bEnd3 cells co-cultured with pre-treated T cells at two time-points (Suppl. Figure 6). Thus, the increased migration of Des-Arg^9^-BK-pre-treated T cells was not attributable to impaired endothelial barrier integrity. Altogether, these findings suggest that PK and Des-Arg^9^-BK signaling promote the migratory functions of activated CD4^+^ T cells, suggesting that the KKS acts as a downstream amplifier of immune cell trafficking responses post-stroke.

### αPK treatment increases circulating T cell frequencies after stroke

**Fig. 6:**
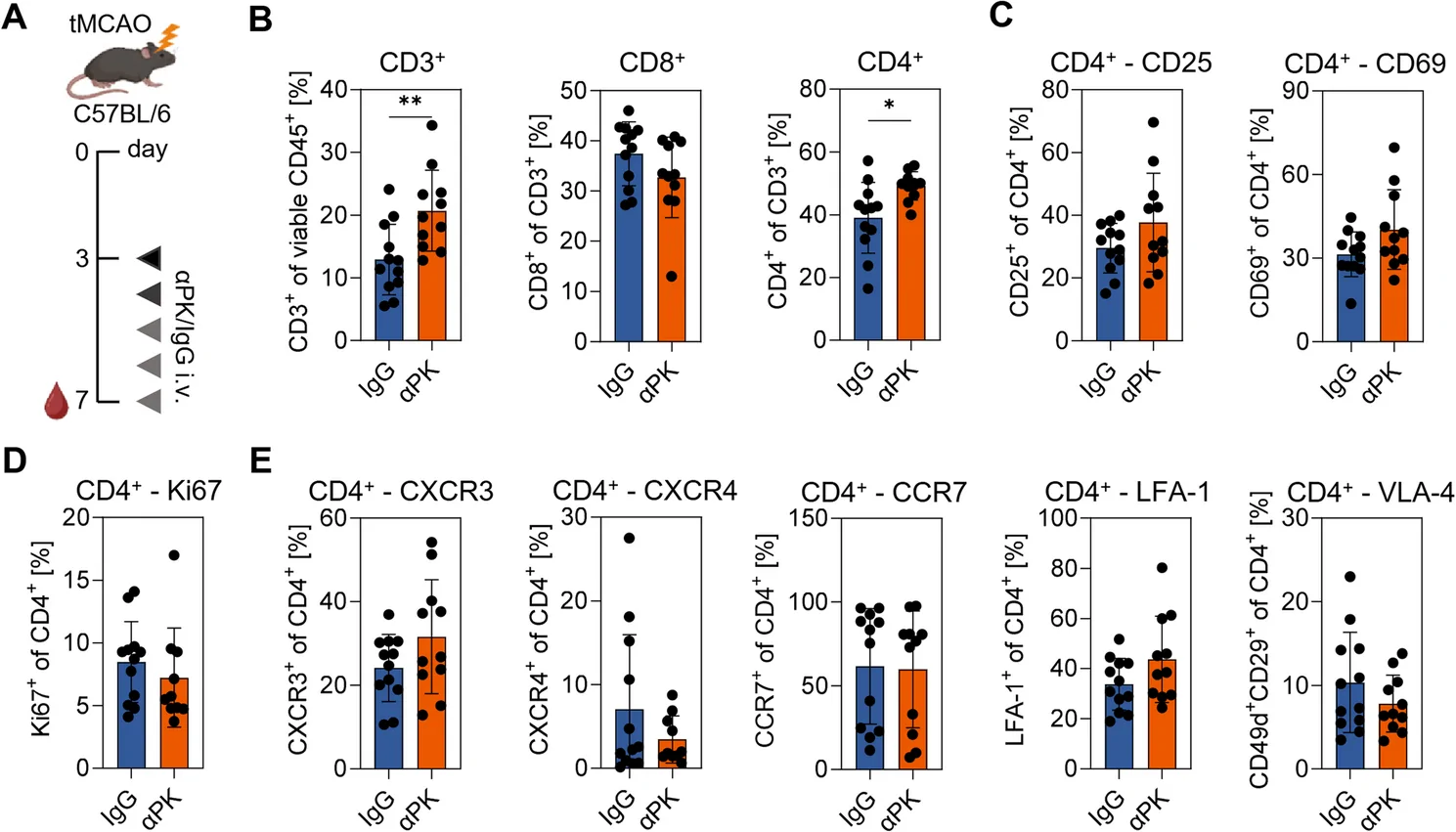
Elevated T cell frequencies in blood of stroke mice treated with plasma kallikrein-neutralizing antibody. **A** C57BL/6 mice were subjected to 60-min transient middle cerebral artery occlusion (tMCAO). Mice were treated with isotype immunoglobulin G (IgG) or a plasma kallikrein-neutralizing antibody (αPK) at days 3 and 4 (10 µg) and days 5 to 7 (5 µg). On day 7, blood was collected and (**B**) T cell subpopulations were analyzed by flow cytometry. CD4^+^ T cells were further examined for expression of (**C**) activation markers, **D** proliferation marker, and **E** adhesion/migration-associated molecules. Data represent three independent experiments (mean ± SD, *n* = 10-12). Statistical significance was determined by unpaired Student’s t-test or Mann-Whitney test, * *p* < 0.05, ** *p* < 0.01. Created with BioRender.com

To evaluate whether the PK- and Des-Arg^9^-BK-mediated in vitro effect on T cell migratory capacity could be prevented by PK inhibition in vivo, we next examined peripheral T cell responses in the blood of stroke mice treated with αPK or IgG control (Fig. 6A, Suppl. Figure 7 A). Given that acute PK blockade has previously been shown to mitigate ischemic injury and thromboinflammation [22], treatment with αPK was initiated at day 3 post-stroke to specifically assess post-acute immune regulation. Despite delayed treatment onset, αPK-treated mice showed increased proportions of circulating CD3^+^ and CD4^+^ T cells compared to IgG-treated controls (Fig. 6B). In contrast, αPK treatment showed no effect on CD4^+^ T cell activation status, proliferative capacity, or expression of adhesion- and migration-associated markers (Fig. 6C-E). Additionally, the frequencies of CD4^+^CD25^+^, CD4^+^CD69^+^, CD4^+^CXCR3^+^, CD4^+^LFA-1^+^ and CD4^+^VLA-4^+^ cells were increased within the total CD45^+^ leukocyte population (Suppl. Figure 7B-C), indicating an overall expansion of the circulating CD4^+^ T cell pool rather than phenotypic modulation at the single-cell level. In line with this, αPK alone did not directly affect CD4^+^ T cell phenotype or functional responses in vitro (Suppl. Figure 8). Redistribution to other organs is also unlikely, since αPK reduced the frequency of CD3^+^ T cells in cervical lymph nodes (LN) and showed a strong trend toward decreased frequencies in the spleen (Suppl. Figure 9). Additionally, PK or Des-Arg^9^-BK treatment lowered apoptosis rates in activated T cells in vitro (Suppl. Figure 10), suggesting that less apoptosis is unlikely to account for the elevated circulating T cell numbers observed in αPK-treated mice.

The lack of detectable changes on a per-cell basis is likely due to the delayed initiation of αPK treatment in vivo (3 days post-stroke), during which circulating T cells were already exposed to elevated BK levels in the acute stroke phase. Supporting this interpretation, in vitro exposure of stimulated splenic CD4^+^CD25^−^ T cells from naïve mice to Des-Arg^9^-BK for 3 days rendered subsequent blockade of B1R signaling with the antagonist Lys-(Des-Arg^9^,Leu^8^)-BK unable to reverse T cell activation, expression of adhesion/migration molecules, and pro-inflammatory cytokine production (Suppl. Figure 11). In contrast, early antagonism of Des-Arg^9^-BK signaling from day 1 markedly reduced the expression of adhesion/migration molecules on CD4^+^ T cells (Suppl. Figure 12). In summary, delayed PK inhibition after ischemic stroke does not interfere with already initiated T cell activation, but leads to an accumulation of CD4^+^ T cells in the peripheral blood, suggesting reduced recruitment into the CNS.

### αPK treatment prevents T cell infiltration into the brain after stroke

**Fig. 7:**
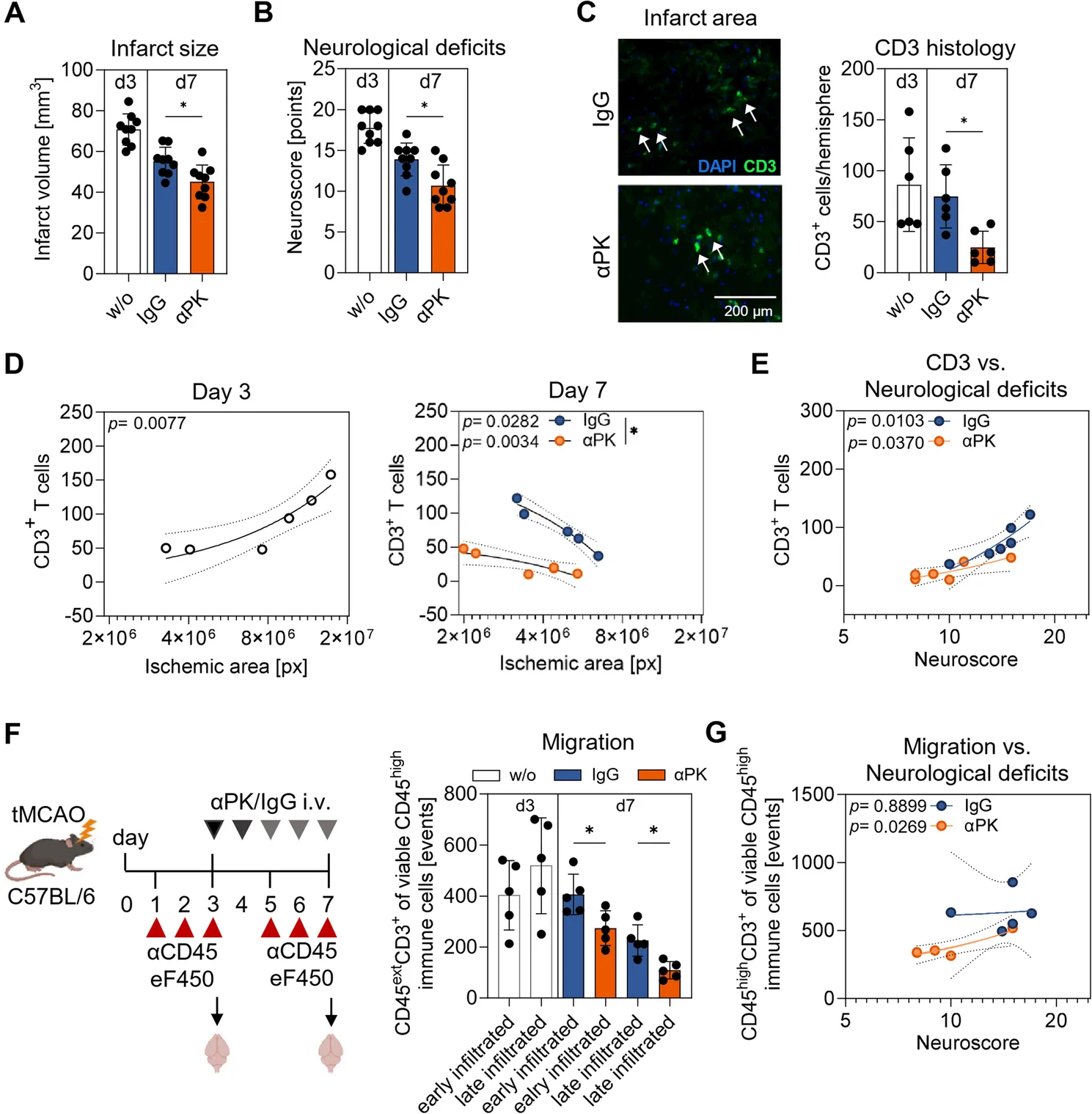
Treatment with plasma kallikrein-neutralizing antibody interferes with T cell infiltration into the brain of stroke mice (**A**) C57BL/6 mice were subjected to 60-min transient middle cerebral artery occlusion (tMCAO). Mice were treated with isotype immunoglobulin G (IgG) or plasma kallikrein-neutralizing antibody (αPK) at days 3 and 4 (10 µg) and days 5 to 7 (5 µg). Infarct sizes were measured at day 3 and 7 using MRI (mean ± SD, *n* = 9). **B** Measurement of neurological deficits at day 3 and 7 after stroke using the Neuroscore (mean ± SD, *n* = 9). **C** Histological analysis of brain CD3⁺ T cells (mean ± SD, *n* = 6). **D** Correlation of infiltrated CD3⁺ T cells with the respective infarct area (px) day 3 (r = 0.8374) and day 7 (IgG: r = 0.7389; αPK: r = 0.9604) after ischemic stroke (*n* = 5-6). **E** Correlation between CD3⁺ T cells and the Neuroscore (IgG: r = 0.8391 αPK: r = 0.7033) (*n* = 6). **F** Mice were injected intravenously with either αPK or IgG control from day 3-7 alongside with αCD45-eFluor450 (αCD45-eF450, i.v.). On day 7, brain T cells were isolated and labeled with an additional αCD45 to distinguish early infiltrated T cells and late infiltrated T cells (mean ± SD, *n* = 5). **G** Correlation of infiltrated CD45^high^CD3⁺ T cells with the Neuroscore (IgG: r = 0.007494; αPK: r = 0.8460) (*n* = 5). Linear associations were assessed by Pearson’s correlation analysis; regression lines with 95 % confidence bands are shown; corresponding p values are indicated. Statistical significance was determined by 2-way ANOVA, unpaired Student’s t-test or Mann-Whitney test, * *p* < 0.05. Created with Biorender.com

Lymphocyte infiltration into the ischemic brain parenchyma is most prominent between days 3 and 7 after stroke [3]. In this study, delayed αPK-treatment led to significantly reduced infarct sizes and was associated with improved neurological outcome at day 7 after stroke compared to IgG control mice (Fig. 7A, B). To further determine whether delayed PK inhibition affects this recruitment phase, we quantified CD3⁺ T cell infiltration into brain tissue collected at days 3 and 7 post-stroke. αPK-treated stroke mice showed a significant reduction in parenchymal CD3⁺ T cell numbers compared to IgG-treated controls at day 7 (Fig. 7C). To exclude infarct size as a confounding factor, we next correlated the infarct area with parenchymal CD3⁺ T cell counts (Fig. 7D). Previous studies in mice with T cell deficiency or T cell depletion show smaller infarct volumes [8, 29]. In our analysis, higher CD3⁺ T cell counts prior to treatment initiation positively correlated with infarct size at day 3 (*r* = 0.8374), while αPK treatment was linked to a significantly lower number of CD3⁺ T cells per infarct volume compared with IgG-treated controls (IgG: *r* = 0.7389; αPK: *r* = 0.9604) at day 7. Higher numbers of parenchymal CD3⁺ T cells were positively correlated with worse neurological outcome in both groups (IgG: *r* = 0.8391 αPK: *r* = 0.7033) at day 7 (Fig. 7E).

To assess whether this reduction resulted from impaired migration of peripherally T cells into the brain rather than changes in local T cell population, circulating immune cells were labeled in vivo with CD45-eFluor450 over 3 consecutive days (days 1–3 or 5–7 post-stroke). Brain-infiltrating immune cells were subsequently isolated and co-stained for CD45 and CD3, to distinguish between early and late infiltrated T cells. At day 7, both early and late recruited CD3⁺ T cell populations were significantly reduced in αPK-treated stroke mice compared with IgG controls (Fig. 7F), indicating that αPK treatment limits T cell migration into the brain. The total lower amount of infiltrated brain CD3⁺ T cells in αPK-treated mice was associated with improved neurological outcome (IgG: *r* = 0.007494; αPK: *r* = 0.8460) at day 7 (Fig. 7G). Collectively, these data suggest that delayed PK inhibition is associated with attenuated T cell infiltration into the ischemic brain tissue, supporting a central role for PK-BK signaling in regulating immune cell trafficking during the subacute phase post-stroke. Altogether, our findings expand the current understanding of the interaction between T cells and the KKS, providing further mechanistic insights into T cell migration and infiltration to the brain parenchyma.

## Discussion

Ischemic stroke triggers a complex neuroimmune response where adaptive immune cells contribute to secondary brain injury and delayed functional decline. In particular, T lymphocytes have emerged as critical mediators of secondary post-stroke neuroinflammation [2]. While components of the KKS have been implicated in BBB disruption and thromboinflammation after stroke, their role in shaping adaptive immune response remains unclear [5, 22, 30]. In fact, whether KKS signaling regulates early T cell function and their migration into the ischemic brain during the subacute phase has not been addressed. Here, we identify the KKS as a modulator of CD4⁺ T cell activation and migration in vitro, thereby supporting a mechanistic link between thromboinflammation and adaptive immunity after stroke.

Post-ischemic inflammation is characterized by a pronounced adaptive immune response. Approximately, up to 50% of brain-infiltrating CD4⁺ and CD8⁺ T cells exhibit an activated effector phenotype within 3 days after experimental stroke, marked by increased CD44 and CD25 expression, and pro-inflammatory cytokine production [31]. While these observations have primarily focused on the CNS, our findings indicate that T cell activation post-stroke is not restricted to the ischemic brain but occurs systemically. Specifically, circulating CD4⁺ T cells in both mice and patients exhibited increased expression of early activation markers, including CD69 and CD25 within 2–4 days after stroke onset. These data indicate a coordinated systemic adaptive immune response conserved across species, highlighting the translational mechanistic relevance of our model.

In addition to activation, circulating CD4⁺ T cells also acquired a distinct trafficking-associated phenotype characterized by enhanced migratory capacity. Increased expression of CCR7 and CXCR3 was detected on peripheral CD4⁺ T cells after experimental stroke. CCR7 is markedly associated with exacerbated neuroinflammatory injury and a worse neurological outcome after stroke [32], promoting the recruitment of multiple T cell subsets into the CNS. Similarly, CXCR3 contributes to T cell migration into the ischemic hemisphere as early as one day post-stroke [33] and is also known to promote firm adhesion of Th1 cells to inflamed brain microvessels in vitro [34]. We further observed increased expression of the integrins VLA-4 and LFA-1 on circulating CD4⁺ T cells in both experimental stroke and patients. These integrins mediate firm adhesion to activated brain endothelium via VCAM-1 and ICAM-1, and transendothelial migration across the BBB after ischemic injury [35]. Consistent with their functional importance, therapeutic blockade of VLA-4 reduces leukocyte infiltration and cytokine production, thereby dampening post-stroke neuroinflammation [36]. The detection of comparable trafficking-associated changes in patient-derived T cells and our preclinical model further supports its clinical relevance, suggesting systemic immune reprogramming as a key mechanistic feature post-stroke.

How vascular and coagulation pathways shape adaptive immune responses during the subacute post-stroke phase remains incompletely defined. The KKS has traditionally been viewed as a regulator of vascular permeability and coagulation with additional roles in endothelial adhesion and leukocyte trafficking [37]. Our data demonstrate an immunomodulatory effect of the KKS at the level of CD4⁺ T cell phenotype and function in vitro. PK enhanced the expression of adhesion and migration markers on CD4⁺ T cells in vitro, whereas serum from αPK-treated stroke mice strongly reduced proliferation and adhesion/migration markers. While PK-dependent upregulation of endothelial adhesion markers such as VCAM-1 and ICAM-1 has been described [23], we extend these findings by indicating that PK simultaneously regulates the corresponding integrin ligands VLA-4 and LFA-1 on CD4^+^ T cells, revealing a coordinated immunomodulatory capacity of the KKS at both endothelial and lymphocyte levels. Therefore, efficient recruitment of adaptive immune cells to the ischemic brain seems to be promoted by direct effects on both endothelial cells and T cells.

Des-Arg⁹-BK, the metabolically active form of BK and major downstream PK modulator, exerted pronounced direct effects on CD4⁺ T cell activation and migration in our study. Activated CD4^+^ T cells expressed both B1R and B2R receptors that are known to be markedly upregulated in the ischemic brain within hours after the ischemic onset [38–40]. In particular, B1R signaling has been implicated in post-ischemic inflammation, BBB disruption and tissue injury. Indeed, pharmacological or genetic inhibition of B1R signaling reduces infarct size, edema formation, and inflammatory cytokine expression in experimental stroke models [38–42]. Here, we demonstrated that Des-Arg^9^-BK, acting through B1R, directly induced a pro-inflammatory, pro-migratory phenotype in CD4⁺ T cells, thereby enhancing adhesion and migration in vitro. These observations are consistent with a broader B1R-dependent motogenic role of kinin signaling in immune cells, as BK has been shown to promote microglial motility and chemotaxis through B1R [43]. Additionally, the CXCL12-CXCR4 axis is a well-established regulator of leukocyte trafficking and has been implicated in cell recruitment and tissue repair in the CNS [44]. Together, these observations support a role for kinin signaling in regulating immune-cell migratory behavior, with our findings specifically identifying Des-Arg^9^-BK/B1R as a pathway capable of directly modulating CD4^+^ T cell adhesion and migration at least in vitro.

T cell infiltration into the ischemic brain is detectable within the first day after stroke, peaks between days 3 and 7, and persists for weeks [3]. Multiple studies demonstrate that T cells contribute to secondary tissue injury and worsen functional outcome, as antibody-mediated depletion of CD4^+^, CD8^+^, and γδ T cells reduces ischemic damage [29]. Therefore, strategies for limiting pathological T cell recruitment to the CNS are essential for stroke recovery.

As a regulator of post-ischemic T cell activation and trafficking, the KKS serves as a promising target to mitigate vascular, thrombotic, and immune-mediated injury after stroke. Genetic deletion of PK reduces intracerebral thrombosis, preserves BBB integrity, and dampens the local inflammatory response in the acute phase after stroke [22]. In the same line, delayed pharmacological inhibition of PK on day 3 - when T cell infiltration is already underway - improves functional outcome, stabilizes the BBB, and reduces T cell numbers in the ischemic hemisphere 7 days post-stroke [5]. Importantly, we extend these observations by mechanistically demonstrating that delayed PK inhibition alters the T cell distribution, increasing circulating T cell frequencies while reducing their accumulation in the ischemic brain, consistent with changes in CNS infiltration dynamics. Indeed, αPK-treated mice showed a significant reduction of CD3⁺ T cell numbers in the ischemic brain area associated with the infarct volume, when compared to non-treated mice. This redistribution of T cells suggests that KKS inhibition modulates adaptive immune cell trafficking rather than inducing systemic immunosuppression, thereby extending its therapeutic relevance beyond vascular protection.

Although our findings provide important insights, several limitations should be considered. First, we did not address the complex interaction between KKS and the renin-angiotensin system (RAS), which counterbalances KKS and also promotes T cell activation, proliferation, and differentiation into pro-inflammatory subtypes [45]. RAS fosters T cell infiltration into inflamed tissue, while comparable mechanisms have also been described for the KKS [46]. Infiltration of T cells into the brain may be facilitated by separate KKS processes at the BBB. PK inhibition promotes BBB stabilization, as reflected by reduced albumin and PK extravasation at day 7 after stroke, together with preservation of the tight-junction proteins claudin-5, occludin, and ZO-1. Improved barrier integrity is associated with significant reduction in T cell and macrophage accumulation within the brain parenchyma after treatment [5]. Therefore, the diminished immune cell infiltration can be explained, at least in part, by reduced BBB disruption and vascular inflammation in addition to direct effects on activated T cells. Importantly, in the same experimental setting, the total number of infiltrating CD45^high^ leukocytes does not differ significantly between IgG- and αPK-treated animals after treatment initiation [5]. This finding, therefore, argues against a generalized reduction in leukocyte entry caused solely by reduced passive leakage and instead suggests a treatment-associated shift in the composition of the infiltrating immune cell populations.

Our findings indicate that Des-Arg^9^-BK, acting predominantly through B1R, can directly modulate CD4^+^ T cell phenotype and function. At the same time, both B1R- and B2R-dependent signaling have been implicated in vascular and inflammatory responses after ischemic stroke [38, 47, 48]. Thus, genetic or pharmacological manipulation of either receptor in vivo would affect multiple cellular compartments and therefore would not provide a sufficiently selective experimental strategy to isolate the contribution of αPK to T cell responses after stroke. In fact, any potential rescue experiments would have multiple off-target effects, constituting a confounding factor in the experimental design. In this study, we also acknowledge that our data may not fully disentangle whether the reduced T cell accumulation observed in vivo is exclusively mediated by a T cell-intrinsic mechanism or, if it is partially influenced by endothelial cells.

As our study primarily focuses on day 3–7 after stroke, it does not allow conclusions regarding potential long-term or permanent alterations in T cell states following ischemic injury. Literature suggests that peripheral T cells may retain their migratory phenotype during the chronic phase following an ischemic event [49]. Using the tMCAO model, T cells were depleted for 4 weeks and subsequently observed reinfiltrating into the brain later, reaching cell numbers comparable to those in control mice [50]. These findings indicate that T cells may still be able to infiltrate in chronic stages, but, in this case, miss the ischemic window to cause additional brain damage. Contrary, other studies conclude that increased T cell numbers in the chronic stage of ischemic stroke models are most commonly attributed to local expansion rather than persistent migration [31, 51]. Continuous T cell activation may have detrimental effects on comorbidities and pathological events in patients. Altogether, since the KKS appears to be chronically altered as well, further experiments should be conducted to elucidate the mechanistic connection in a chronic scenario. Additionally, exploring the role of different pro-inflammatory and anti-inflammatory CD4^+^ T cell subsets would indeed expand our current understanding of the KKS-T cell axis particularly in relation to long-term recovery after stroke.

In summary, we establish a previously unrecognized functional relationship between KKS signaling and CD4^+^ T cell responses that links thrombinflammation to adaptive immunity after stroke. Our in vitro findings indicate that KKS signaling modulates processes involved in CD4^+^ T cell activation and trafficking, whereas the in vivo effects are likely to involve both endothelial- and T cell-associated mechanisms. Pharmacological inhibition of PK is associated with reduced T cell accumulation in the ischemic hemisphere, without evidence of systemic immunosuppression. Collectively, these observations position the KKS as an important regulator of neuroimmune interactions and support its potential as a therapeutic target to modulate detrimental T cell-associated responses after stroke.

## Methods

### Collection of human blood samples

Peripheral blood samples were obtained from patients after ischemic stroke together with non-stroke controls enrolled in a study approved by the local human ethics committee of the University Hospital Essen (reference 21-10271-BO) [52]. All participants or their legal representatives provided written informed consent prior to inclusion. Sample collection and handling were conducted in accordance with the Declaration of Helsinki and institutional guidelines for human biomedical research. Ischemic stroke was diagnosed based on clinical evaluation and confirmed by neuroimaging. Clinical and demographic data were recorded for all patients including age, sex, comorbidities (e.g., hypertension, diabetes, atrial fibrillation), pre-admission medication, National Institutes of Health Stroke Scale (NIHSS) score at the time of sampling, and received acute treatments (intravenous thrombolysis and/or thrombectomy). Blood samples from stroke patients were collected at two predefined time points after the ischemic event: an acute cohort (2–4 days post-stroke) and a subacute cohort (5–7 days post-stroke). In both, patients were matched for sex, age, and treatment modality (Supplemental Table 1). Patients receiving immunomodulatory medication prior to or during sample collection were excluded, as were individuals with chronic inflammatory, autoimmune, or malignant diseases. Healthy control participants had no history of cerebrovascular disease, chronic inflammatory or autoimmune disorders, or active infection at the time of sampling. Healthy controls consisted of male and female patients with no relevant comorbidities and minimal premedication.

### Isolation of human peripheral blood mononuclear cells

Peripheral blood was collected into EDTA-coated tubes. Blood samples from individual donors were pooled into 50 ml conical tubes and gently homogenized by repeated pipetting with a serological pipette. The pooled blood was diluted 1:1 with phosphate-buffered saline (PBS; pH 7.4) and carefully mixed by gentle pipetting. Peripheral blood mononuclear cells (PBMCs) were isolated by density gradient centrifugation using Ficoll-Paque™ (GE Healthcare, Chicago, USA). Briefly, 15 ml of Ficoll-Paque was added to a 50 ml conical tube and approximately 20–30 ml of the diluted blood sample was carefully layered on top using a serological pipette, while maintaining a clear phase boundary avoiding mixing both layers. Samples were then centrifuged at 1000 × g for 25 min at 25 °C, with low acceleration and no brake to allow gradient formation. After centrifugation, the mononuclear cell layer at the plasma–Ficoll interface was carefully collected using a glass pipette and transferred to a new 50 ml tube containing 20 ml of cold PBS. Cells were washed with cold PBS, followed by centrifugation at 500 × g for 10 min at 4 °C. The supernatant was discarded and the cell pellet was thoroughly resuspended in 1 ml PBS followed by counting using a hemocytometer. To remove residual platelets and debris, cells were washed again with PBS and centrifuged at 300 × g for 10 min at 4 °C. The final cell pellet was resuspended in recovery cell culture freezing medium (Gibco, Darmstadt, Germany), aliquoted, and placed in a controlled-rate freezing container (Mr. Frosty™, Thermo Fisher Scientific, Langenselbold, Germany). Samples were stored at -80 °C for at least 4 h and up to 1 week before transfer to liquid nitrogen for long-term storage.

### Experimental animals

In this study, adult male and female C57BL/6J mice (Charles River Laboratories, Sulzfeld, Germany and Envigo, Horst, Netherlands) were included. For in vivo experiments, male mice were used to ensure comparability with previously published data [5]. For in vitro assays, both female and male mice were used as donors to obtain primary cell preparations, with a predominance of females in the experimental cohort. No differences were observed between male- and female-derived T cells, at least for the expression of migration- and adhesion-associated molecules (Suppl Fig. 5). Animals were group-housed (up to 5 per cage) in individually ventilated cages under controlled environmental conditions (22 °C, 55 ± 5% humidity and 12 h light/12 h dark circadian rhythm), with *ad libitum* access to food and water. All animal procedures were approved by the local state authorities (Landesamt für Verbraucherschutz und Ernährung NRW, LAVE) and conducted in agreement with the German Animal Welfare Act (German Ministry of Agriculture, Health and Economic Cooperation).

### Ischemia model: transient middle cerebral artery occlusion

Focal cerebral ischemia was induced by 60 min of tMCAO as previously described [5]. Mice were anaesthetized with 4% isoflurane (Piramal Critical Care, Vorschooten, Netherlands) in 100% O_2_ for 2–4 min using a small animal anesthesia system (EZ-7000, World Precision Instruments, Friedberg, Germany), followed by maintained anesthesia with ~ 2% isoflurane. Body temperature was maintained at 37 °C throughout the procedure using a feedback-controlled warming system (ATC-2000, World Precision Instruments, Friedberg, Germany). Following a midline skin incision at the throat, the right common carotid artery (CCA) and the external carotid artery (ECA) were exposed. The CCA was ligated temporarily, while the ECA was permanently ligated. The internal carotid artery (ICA) was temporarily occluded using a vascular clip (Fine Science Tools Inc., Foster City, CA, USA). For induction of cerebral ischemia, a silicon rubber-coated monofilament (Doccol, Corporation, USA) was introduced via the ECA and advanced into the ICA until occluding the origin of the right middle cerebral artery (MCA). The intraluminal suture was left in place for 60 min, before the filament was removed to allow for reperfusion. The temporary ligation of the CCA was then released, before suturing the incision. Mice were excluded from final analyses if they developed intracranial bleedings, hemorrhagic transformation, or showed no detectable infarct. Of the animals subjected to tMCAO (*n* = 70), 68 were included in the final analysis. Two mice were excluded for one or more of the reasons stated above. Neurological deficits were evaluated using the neuroscore as previously described [53].

### MRI scanning and analysis

To ensure comparable baseline conditions between treatment groups and to exclude possible intracerebral bleeding, MRI was performed at 3 days post ischemia (dpi) using a 3 Tesla MRI unit (Biograph mMR, Siemens Healthineers, Germany). A commercially available dual channel surface coil designed for examining mice (Rapid Biomedical GmbH, Rimpar, Germany) was used. The MRI protocol included a coronal T2-weighted turbo spin-echo sequence for infarct delineation (resolution 0.2 × 0.2 × 0.8 mm³, 15 slices, TE = 105 ms, TR = 1660 ms, TA = 3:47 min) and a susceptibility weighted coronal T2-weighted gradient echo sequence (SWI, 0.1 × 0.1 × 0.2 mm³, 40 slices, TE = 20.6 ms, TR = 38 ms, TA = 6:01 min) for detecting intracerebral hemorrhage. Anatomical localization was refined using short T1-weighted TSE sequences along each axis (0.2 × 0.2 × 1 mm³, 7 slices, TA = 17s). All MRI measurements were conducted under constant anesthesia induced by intraperitoneal injection of ketamine (0.1 mg/g) and xylazine (0.005 mg/g). The T2-weighted TSE sequences were used for planimetric evaluation of edema corrected infarct volume using the indirect measurement technique as well as evaluation of edema formation. Calculation of infarct size was performed using ImageJ (U.S. National Institutes of Health, Bethesda, USA) according to the following equation: V_indirect_ [mm^3^] = [V_infarct_× (1-(V_ih_ – V_ch_)/V_ch_)] × 1.6, where V_infarct_ represents the hyperintense lesion area, V_ih_ the volume of the ipsilateral hemisphere, V_ch_ the volume of the contralateral hemisphere, and 1.6 represents the interslice spacing [mm]. Brain edema was calculated according to: Edema [%] = V_total_ × 100/(V_ch_ × 2) − 100, where V_total_ represents the total brain volume. Quantification was performed for every second image, and values were summed up to obtain total infarct size or edema formation respectively.

### Pharmacological intervention

Starting 3 dpi, mice were assigned to treatment groups after MRI assessment. Animals received either a PK-neutralizing antibody (αPK 1B; #GTX21006, GeneTex, Irvine, USA) or a mouse IgG1 isotype control antibody (#400165, BioLegend, San Diego, USA), administered via intravenous (i.v.) injection. Mice received 10 µg of the respective antibody on 3 and 4 dpi, followed by 5 µg on 5, 6 and 7 dpi.

### Flow cytometry

The following fluorochrome-conjugated antibodies were used for flow cytometry analyses. For murine cells: ɑCD3, ɑCD4, ɑCD8, ɑCD69, ɑCD25, ɑCD44, ɑCXCR3, ɑCXCR4, ɑKi67, ɑIFN-γ, ɑTNF-ɑ (BD Biosciences, Heidelberg, Germany); ɑCD29, ɑCD49d, ɑLFA-1, ɑCCR7, and ɑIL-2 (BioLegend, San Diego, USA). For human cells: ɑCD3, ɑCD4, ɑCD69, ɑCD25, ɑCD29, ɑCXCR3, ɑCXCR4, ɑLFA-1 (BD Biosciences, Heidelberg, Germany); and ɑCD49d (BioLegend, San Diego, USA). Dead cells were excluded using the Fixable Viability Dye eFluor 780 (FvD; eBioscience, Thermo Fisher Scientific, Langenselbold, Germany). For surface staining, cells were centrifuged at 300 x g for 5 min at 4 °C, followed by incubation with the respective surface antibody mix for 10–15 min at 4 °C in the dark. Where indicated, intracellular staining was performed after surface labeling. For detection of the proliferation marker Ki67, the Foxp3/transcription factor staining kit (eBioscience, Thermo Fisher Scientific, Langenselbold, Germany) was used according to the manufacturer’s instructions. Briefly, cells were fixed in a fixation/permeabilization buffer for 1.5 h at 4 °C in the dark. After centrifugation at 300 x g for 5 min at 4 °C, cells were stained with the respective intracellular antibodies for 30 min at 4 °C. Cell proliferation was assessed using the Proliferation Dye eFluor 670 (eBioscience, Thermo Fisher Scientific, Langenselbold, Germany).

For intracellular staining of INF-γ, TNF-ɑ, and IL-2, cells were stimulated for 4 h at 37 °C with IMDMcomplete containing 10 ng/ml phorbol-12-myristate-13-acetate, 5 µg/ml brefeldin A and 1 µg/ml ionomycin. Then, cells were centrifuged at 300 x g for 5 min at 4 °C and subjected to surface staining as described above. Afterwards, cells were fixed in 2% paraformaldehyde for 15 min at 4 °C, then permeabilized in 0.1% Nonidet P-40 (Sigma-Aldrich, Taufkirchen, Germany) in H_2_O at room temperature and finally stained with the respective intracellular antibodies for 30 min at 4 °C. Between each step, cells were washed with PBS.

Apoptosis was assessed using the Annexin V Apoptosis Detection Kit APC (eBioscience, Thermo Fisher Scientific, Langenselbold, Germany) according to manufacturer’s instructions. Following standard surface marker staining, cells were washed twice with PBS and incubated in 1 x Annexin V binding buffer containing Annexin V and 7-AAD for 15 min at room temperature in the dark. Subsequently, 1 x Annexin binding buffer was added, cells were centrifuged at 300 x g for 5 min at 4 °C, and resuspended in Annexin V binding buffer.

### Collection of mouse tissue samples after tMCAO

Mice subjected to tMCAO surgery and allocated to either treatment or control groups were sacrificed on day 3 or 7 post-ischemia. Animals were anaesthetized via intraperitoneal injection of ketamine (0.1 mg/g) and xylazine (0.02 mg/g), and transcardially perfused with PBS. Following decapitation, brains were carefully removed from the skull, avoiding damage to the cortical surface. Brains were immediately placed in an ice-cold stainless-steel brain matrix (Zivic Instruments, Pittsburgh, USA) and coronally sectioned into four 2 mm slices using razor blades. Tissue sections were then processed appropriately for subsequent use. Peripheral blood was collected immediately prior to sacrifice. Blood samples were either used for leukocyte isolation or left at room temperature for clotting (for at least 30 min) and then centrifuged at 2500 x g for 15 min. Serum was carefully transferred to a separate tube and stored at -80 °C until further use. For preparation of leukocyte single-cell suspensions, freshly collected blood was immediately transferred into 1 ml of erythrocyte lysis buffer and gently inverted to prevent clot formation. Following 2 min incubation at room temperature, samples were centrifuged at 200 x g for 10 min at 4 °C. Erythrocyte lysis was repeated until a clean leukocyte pellet was obtained. The final cell pellet was resuspended with PBS supplemented with 2% FCS and 2 mM EDTA. Spleens and cervical LNs were isolated and mechanically dissociated through a 70 μm cell strainer. Cervical LNs were processed using PBS supplemented with 2% FCS and 2 mM EDTA, whereas spleens were dissociated in erythrocyte lysis buffer followed by PBS supplemented with 2% FCS and 2 mM EDTA.

### Protein quantification by bicinchoninic acid assay

Total protein concentration was determined using the Pierce BCA Protein Assay Kit (Thermo Fisher Scientific, Langenselbold, Germany) according to the manufacturer’s instructions. Briefly, tissue homogenates were diluted 1:20 in distilled water prior to analysis. Samples and bovine serum albumin (BSA) standards were analyzed in duplicates in a 96-well microplate. A standard curve was generated using BSA standards ranging from 0 to 2000 µg/ml. Twenty-five microliters of each standard or diluted sample were pipetted into the wells, followed by the addition of 200 µl freshly prepared BCA working reagent. Plates were sealed and incubated for 30 min at 37 °C. Absorbance was measured at 560 nm using an Infinite^®^ F200 PRO microplate reader (Tecan, Männedorf, Switzerland). Protein concentrations were calculated from the BSA standard curve and corrected for the sample dilution factor.

### Splenic T cell isolation

To obtain CD4^+^CD25^−^ T cells from the spleen of C57BL/6 mice, the organ was flushed with 3 ml of erythrocyte lysis buffer and mechanically dissociated through a 70 μm cell strainer. Lysis was stopped by adding 12 ml PBS supplemented with 2% FCS and 2 mM EDTA. The resulting cell suspension was then centrifuged at 300 x g for 10 min at 4 °C. CD4^+^ T cells were isolated by using the CD4^+^ T cell isolation kit (Miltenyi Biotec, Bergisch Gladbach, Germany) according to the manufacturer’s specifications. To deplete regulatory T cells, a biotin-conjugated anti-mouse CD25 antibody (BD Biosciences, Heidelberg, Germany) was included during the magnetic separation process. Purified CD4^+^CD25^−^ T cells were centrifuged at 300 x g for 10 min at 4 °C and resuspended in Iscove’s Modified Dulbecco’s Medium (IMDM, Gibco, Thermo Fisher Scientific, Langenselbold, Germany) supplemented with 10% FCS, 100 µg/ml Streptomycin, 100 U/ml Penicillin and 25 µM ꞵ-mercaptoethanol (IMDMcomplete) for further processing.

### In vitro assays

Splenic CD4^+^CD25^−^ T cells from naive C57BL/6 mice were isolated as described above. 2.5 × 10^5^ T cells were resuspended in IMDMcomplete and plated in 96-well flat-bottom plates. Experimental treatments were applied individually or in combination, including: serum from ɑPK- or IgG-treated tMCAO mice, human PK (#HPKa 1303, Enzyme Research Laboratories, South Bend, USA), αPK 1B (#GTX21006, GeneTex, Irvine, USA), [Des-Arg9]-Bradykinin (acetate) (#HY-P0298A, MedChemExpress, Monmouth Junction, USA) and Lys-(Des-Arg9,Leu8)-Bradykinin (#HY-P4676, MedChemExpress, Monmouth Junction, USA). To provide an activating T cell receptor signal, cells were stimulated with 1 µg/ml plate-bound anti-mouse CD3e and 1 µg/ml soluble anti-mouse CD28 (both BD Biosciences, Heidelberg, Germany). Cells were incubated at 37 °C with 5% CO_2_ and analyzed by flow cytometry after 24 h, 48 h, or 72 h. Supernatants harvested after 72 h were used for cytokine analysis by Luminex. To identify proliferating cells after 72 h incubation, cells were labelled with the Proliferation Dye eFluor 670 (eBioscience, Thermo Fisher Scientific, Langenselbold, Germany) prior to seeding the cells into the 96-well plate. Briefly, cells were washed twice with PBS, resuspended in 2 ml of PBS at room temperature to a concentration of 1 × 10^7^ cells, and incubated with the Proliferation Dye eFluor 670 in the dark at 37 °C for 10 min. Staining was stopped with 4 ml of ice-cold PBS followed by 5 min incubation on ice. Cells were then washed twice with IMDMcomplete and resuspended before plating.

### Cytokine quantification

Cytokine levels in cell culture supernatants were quantified using a polystyrene bead-based Luminex Assay (BIO-Techne GmbH, Minneapolis, USA) according to the manufacturer’s instructions. Analysis was performed on a Luminex 200 system with IS software ensuring standardization and reproducibility across samples.

### Transwell migration assay

To evaluate the effects of PK and Des-Arg^9^-BK on T cell migratory capacity, splenic CD4^+^CD25^−^ T cells from naive C57BL/6 mice were stimulated with ɑCD3/ɑCD28 in the presence of 0, 0.1, 1, or 10 µg/ml PK or Des-Arg^9^-BK for 48 h, as described above. T cells were then harvested, pooled by the treatment group, washed twice with IMDM supplemented with 0.1% BSA at 300 x g for 10 min at 4 °C, and resuspended in IMDM supplemented with 0.1% BSA. Transwell inserts (5 μm pore size; Corning, Sigma-Aldrich, St. Louis, USA) were coated with 6.5 µg/ml fibronectin for 60–90 min at 37 °C and washed twice with PBS. A total of 0.6 × 10^6^ T cells were added per insert. Lower chambers contained IMDM plus 0.1% BSA alone (undirected migration) or supplemented with 100 ng/ml recombinant rh/feCXCL12/SDF-1β (BIO-Techne GmbH, Minneapolis, USA) for directed migration. After 4 h at 37 °C, migration was stopped by adding 0.5 M EDTA to the lower chamber for 10 min. All migrated T cells in the lower chamber were collected, centrifuged twice at 500 x g for 10 min at 4 °C, and counted using a Neubauer chamber. Migration was expressed as fold change relative to undirected migration of untreated control cells (0 µg/ml PK/Des-Arg^9^-BK).

### Transwell adhesion and migration assay

Splenic CD4^+^CD25^−^ T cells from naive C57BL/6 mice were stimulated with ɑCD3/ɑCD28 in the absence or presence of 10 µg/ml Des-Arg^9^-BK for 48 h, as described in above. T cells were then harvested, pooled by the treatment group, washed twice with IMDM supplemented with 0.1% BSA at 300 x g for 10 min at 4 °C, and resuspended in IMDM supplemented with 0.1% BSA. Transwell inserts (8 μm pore size; Corning, Flacon, Sigma-Aldrich, St. Louis, USA) were washed three times with PBS before seeding 0.5 × 10^5^ bEnd3 endothelial cells per insert in IMDMcomplete. After 48 h, a confluent monolayer was established, and inserts were washed with IMDM supplemented with 0.1% BSA. Subsequently, 0.5 × 10^6^ T cells were added to each insert. Lower chambers contained IMDM with 0.1% BSA alone (undirected migration) or supplemented with 100 ng/ml rh/feCXCL12/SDF-1β (directed migration). After 4 h at 37 °C, transwells were fixed with 4% PFA for 15 min and all cells without contact to the endothelium were washed away by PBS. Next, inserts were blocked with 2% FCS for 40 min and washed again. T cells were stained with FITC-conjugated ɑCD3 (Miltenyi Biotec, Bergisch Gladbach, Germany) and nuclei counterstained with Hoechst (Invitrogen, Thermo Fisher Scientific, Langenselbold, Germany) for 1 h. After final PBS washes, T cells that adhered on top of the endothelium and T cells that transmigrated and still were in contact to the lower side of the endothelium were quantified by confocal fluorescence microscopy using the Elyra PS.1 microscope (Carl Zeiss AG, Oberkochen, Germany). Data are presented as fold change relative to undirected adhesion/migration of untreated control cells.

### Transendothelial electrical resistance measurement

Transwell adhesion and migration assays containing Des-Arg^9^-BK-pre-treated T cells and bEnd3 cells were set up as described above. Lower chambers contained IMDM with 0.1% BSA, supplemented with 100 ng/ml rh/feCXCL12/SDF-1β. After 2.5 and 4 h at 37 °C, the TEER of the endothelial cell layer was measured using the Millicell ERS-2 Electrical Resistance System (Merk Millipore, Darmstadt, Germany). TEER was calculated using the following equation: Resistance = (measured value [Ω] - blank [Ω]) * 0.3 [cm^2^].

### Kinin receptor PCR

Splenic CD4^+^CD25^−^ T cells from naive C57BL/6 mice were cultured in IMDMcomplete medium and either stimulated with ɑCD3/ɑCD28 or left unstimulated for 48 h. Total RNA from T cells and bEnd3 cells (positive control) was isolated using the RNeasy Mini Kit (Qiagen, Hilden, Germany) according to the manufacturer’s instructions. RNA concentration and purity were measured using the NanoDrop 1000 Spectrophotometer (PEQLAB Biothechnologie GmbH, Erlangen, Germany) and analyzed by the ND-1000 software. For complementary DNA (cDNA) synthesis, 1–2 µg RNA was diluted in nuclease-free water to 13 µl and mixed with 0.5 µl Oligo(dT) primer (0.5 µg/µl, Invitrogen, Thermo Fisher Scientific, Langenselbold, Germany) and 0.5 µl random hexamers (3 µg/ml, Invitrogen, Thermo Fisher Scientific, Langenselbold, Germany). Samples were incubated at 70 °C for 10 min and cooled to 42 °C on ice. Reverse transcription was performed by adding 1 µl dNTPs (10 mM), 4 µl MML V-RT 5x buffer (Promega, Fitchburg, USA), and 1 µl MMLV-reverse transcriptase (H-) point mutant (200 U/µl, Promega, Fitchburg, USA) followed by incubation at 42 °C for 1 h. Enzyme inactivation was conducted at 95 °C. Successful cDNA synthesis was confirmed by semi-quantitative PCR of the housekeeping gene *ribosomal protein 9* (*Rps9*, RPS9_F: 5´-CTGGACGAGGGCAAGATGAAGC-3´, RPS_R: 5´-TGACGTTGGCGGATGAGCACA-3´). PCR amplification of *kinin receptor 1* (*B1r*, B1R_F: 5´-CCATAGCAGAAATCTACCTGGCTAAC-3´, B1R_R: 5´-GCCAGTTGAAACGGTTCC-3´) and *kinin receptor 2* (*B2r*, B2R_F: 5´-CTGGCAGCGGCGGACCTCATC-3´, B2R_R: 5´-CCCGGCACAACACCTCTCCAAACA-3´) was then performed. PCR products were visualized by agarose gel electrophoresis on 1% gels stained with Midori Green (NIPPON Genetics EUROPE GmbH, Düren, Germany), using the Gene Ruler 100 bp Plus DNA ladder.

### Migration in vivo assessment

To distinguish circulating from tissue-infiltrating leukocytes, mice were intravenously injected with 3 µg of anti-CD45-eFluor450 on 2 consecutive days starting from either 1–3 or 5–7 days after stroke. A final dose of 3 µg αCD45-eFluor450 was administered intravenously 4 h prior to sacrifice and perfusion. Animals were then transcardially perfused with 20 ml of ice-cold PBS. Brains were then isolated and stored in 10 ml of ice-cold PBS, followed by bisecting into hemispheres for further processing. Tissue dissociation was performed according to the manufacturer’s protocol for inflamed neural tissue using the Multi Tissue Dissociation Kit 1 (Miltenyi Biotec, Bergisch-Gladbach, Germany). Cell suspensions were subjected to debris removal and gradient centrifugation. Prior to flow cytometry analysis, cells were stained with anti-CD45-PE, anti-CD3-FITC, and propidium iodide to assess cell populations and viability. Cells were analyzed using a MACSQuant Analyzer Flow Cytometer (Miltenyi Biotec, Bergisch-Gladbach, Germany).

### Histological analysis

PBS-fixed tissue brain sections were air-dried and fixed in formalin for 10 min at room temperature. Slides were then washed in TBST (TBS + 0.05% Tween-20) and blocked for 1 h in TBST containing 5% normal donkey serum, 5% normal goat serum, and 1% BSA. Sections were incubated with a rat anti-CD3 primary antibody (1:200, Bio-Rad, Feldkirchen, Germany) diluted in blocking buffer for 24 h at 4 °C. Following washing, sections were incubated 1 h at room temperature with an AF488-conjugated anti-rat secondary antibody (1:500) (Invitrogen, Darmstadt, Germany, USA) and DAPI (Sigma-Aldrich, Munich, Germany) counterstain. After final washes, slides were mounted with Mowiol/Dabco. CD3⁺ T cell numbers were quantified using ImageJ software. Images were converted to 8-bit format, channel-split, and merged for analysis (DAPI: blue; CD3: green). Double-positive cells were manually counted using the CellCounter plugin with a grid overlay. For infarct area analysis, images were normalized for pixel density using IrfanView and subsequently analyzed in ImageJ. Infarct regions were selected using the freehand tool, measured in pixel squared (px²), and correlated with immune cell infiltration.

### Statistical analysis

All graphs and statistics were performed with GraphPad Prism 10 (GraphPad Software, LA Jolla, USA). Data was tested for normality using D’Agostino & Pearson omnibus or Shapiro-Wilk normality test. In case of normality paired or unpaired Student’s *t*-test for two samples, or RM one-way ANOVA (if applicable two-way ANOVA followed by Sidák’s multiple comparisons test or Tukey’s multiple comparisons test) or Mixed-effects analysis for multiple samples were used to calculate statistically significant differences. If data sets did not pass normality, non-parametric Mann-Whitney test or Wilcoxon test for two samples, or Friedman test or Kruskal-Wallis test for multiple samples were used. Simple linear regression analysis was used to assess linear associations, with the correlation coefficient (*r*) and corresponding *p* values reported. Regression lines are shown with 95% confidence bands. The respective test used to calculate statistically significant differences is described in the figure legends. Biological replicates with mean (± SD) are depicted as one dot. Statistical significance was considered as *p*-value of 0.05 or less (* *p* < 0.05, ** *p* < 0.01, *** *p* < 0.001, **** *p* < 0.0001).

## Supplementary Information

Supplementary Material 1.

Supplementary Material 2.

## Data Availability

The data that support the findings of this study are available from the corresponding author upon reasonable request.

## Abbreviations

αPK: PK-neutralizing antibody
B1R: Kinin receptor B1
B2R: Kinin receptor B2
BBB: Blood-brain barrier
BCA: Bicinchoninic Acid assay
BK: Bradykinin
BSA: Bovine serum albumin
CCA: Common carotid artery
cDNA: Complementary DNA
CNS: Central nervous system
DAMPs: Damage-associated molecular patterns
Des-Arg^9^-BK: Des-Arg^9^-bradykinin
dpi: Days post ischemia
ECA: External carotid artery
EDTA: Ethylenediaminetetraacetic acid
FCS: Fetal calf serum
FvD: Fixable viability dye
FXII: Factor XII
HC: Healthy control
HMWK: High-molecular-weight kininogen
ICA: Internal carotid artery
IgG: Immunoglobulin-G
IMDM: Iscove’s Modified Dulbeccos’s Medium
IMDMcomplete: IMDM supplemented with 10% FCS, 100 µg/ml Streptomycin, 100 U/ml Penicillin and 25 µM β-mercaptoethanol
KKS: Kallikrein-kinin system
LAVE: Landesamt für Verbraucherschutz und Ernährung NRW
LN: Lymph node
MBMECs: Murine brain microvascular endothelial cells
MCA: Middle cerebral artery
NIHSS: National Institutes of Health Stroke Scale
NK: Natural killer
PBMCs: Peripheral blood mononuclear cells
PBS: Phosphate-buffered saline
PI: Propidium iodide
PK: Plasma kallikrein
px^2^: Pixel squared
RAS: Renin-angiotensin system
RPS9: Ribosomal protein S9
SDF-1β: Stroma cell-derived factor-1β
TBST: TBS + 0.05 % Tween-20
TEER: Transendothelial electrical resistance
Th: Helper T cells
Th1: Type 1 helper T cells
tMCAO: Transient middle cerebral artery occlusion
