# Supplementary material for: The kallikrein-kinin system modulates CD4+ T cell function and contributes to T cell migration into the brain after stroke
Source: J Neuroinflammation. 2026 Sep 5;23:296. doi: 10.1186/s12974-026-04034-4 (PMC13545774; doi:10.1186/s12974-026-04034-4)
Supplement: Supplementary file 1 [file 12974_2026_4034_MOESM1_ESM.docx]

**Supplemental information for**

**The kallikrein-kinin system modulates CD4^+^ T cell function and contributes to T cell migration into the brain after stroke**

Nadine Gausmann^1,*,§^, Steffen Haupeltshofer^2,3,*^, Rebecca D. Szepanowski^2^, Sina Luppus^1^, Egor Dzyubenko^2^, Jasmin Bahr^2^, Stine Mencl^2^, Janine Gronewold^2^, Dirk M. Hermann^2^, Michael Forsting^4^, Cornelius Deuschl^4^, Jan Buer^1^, Astrid M. Westendorf^1^, Christoph Kleinschnitz^2^, Ana I. Casas^2,5,#^, Wiebke Hansen^1,#^

^1^Institute of Medical Microbiology, University Hospital Essen, Essen, Germany

^2^Department of Neurology and Center for Translational Neuro- and Behavioral Sciences (C-TNBS), Clinic for Neurology, University Hospital Essen, Essen, Germany

^3^Translational Neurology Group, Department of Clinical Science, Lund University, Lund, Sweden

^4^Institute of Diagnostic and Interventional Radiology and Neuroradiology, University Hospital Essen, Essen, Germany

^5^Department of Pharmacology and Personalised Medicine, Faculty of Health, Medicine and Life Sciences, Maastricht University, Maastricht, The Netherlands

*Both first authors contributed equally

^#^Both last authors contributed equally

^§^Corresponding author: nadine.gausmann@uk-essen.de

**Supplemental Figures**

**Fig. S1.**

**Supplemental Fig.1: The plasma kallikrein-kinin pathway and candidate receptors on T cells.** The KKS links thrombotic and inflammatory pathways following ischemic stroke. (1) Under pathophysiological conditions, platelets are activated upon endothelial damage, leading to the release of polyphosphates, activating FXII. (2) Alternative pathways resulting in the activation of FXII include direct contact to collagen exposed by damaged endothelium and contact to a complex of HMWK and PPK, which is bound to collagen on the site of injury. (3) FXIIa activates FXI, (4) subsequently activating thrombin. (5) Thrombin itself is able to facilitate the enzymatic cleavage of fibrinogen to fibrin, thereby forming and stabilizing the thrombus. (6) FXIIa initiates the contact-kinin pathway, ultimately cleaving PPK into PK. Reciprocally, PK activates FXII, establishing a positive feedback loop. (7) PK subsequently cleaves HMWK into the pro-inflammatory peptide hormone BK. (8) Once BK binds to its endothelial kinin receptors 1 and 2, several inflammatory signaling cascades are initiated and result in breakdown of tight junction proteins and the formation of edema. (9) Ultimately, BK also binds to B1R and B2R expressed on T cells, whereas the receptor mediating direct PK effects on T cells remains unknown. Abbreviations: B1/2R, bradykinin receptor 1/2; BK, bradykinin; FXI, factor XI; FXII, factor XII; kallikrein-kinin system, KKS; HMWK, high-molecular-weight kininogen; PK, plasma kallikrein; PPK, plasma prekallikrein; polyP, polyphosphates. Adapted from [1]. Created with BioRender.com

**Fig. S2.**

**Supplemental Fig. 2: Effect of stroke on human peripheral blood T cells and corresponding gating strategy.** Peripheral blood was collected from healthy controls (HC) and ischemic stroke patients 2-4 and 5-7 days after onset. Peripheral blood mononuclear cells (PBMCs) were isolated and T cells were analyzed by flow cytometry. (A) Representative gating strategy for blood T cell populations. At first, cells were gated for leukocytes and doublets were excluded. After gating for viable CD45^+^ cells, CD45^high^CD3^+^ T cells were selected and divided into CD8^+^ T cells and CD4^+^ helper T cells. CD4^+^ T cells were analyzed for expression of activation and adhesion/migration-related molecules, shown exemplary for CD69. (B) Frequencies of CD4^+^ T cells and (C) expression of CD69, CXCR3, LFA-1, VLA-4, CD25, and CXCR4 on CD4^+^ T cells (mean ± SD, *n* = 11). Statistical significance was determined by RM one-way ANOVA or Kruskal-Wallis test, * *p* < 0.05, ** *p* < 0.01, *** *p* < 0.001.

**Fig. S3.**

**Supplemental Fig. 3: Gating strategy for the determination of T cells in the blood of tMCAO mice.** C57BL/6 mice were subjected to 60-min transient middle cerebral artery occlusion (tMCAO) or left untreated. Blood was collected on day 3 and analyzed by flow cytometry. Shown is the representative gating strategy for blood T cell populations. At first, cells were gated for leukocytes and doublets were excluded. After gating for viable CD45^+^ cells, CD45^high^CD3^+^ T cells were selected and divided into CD8^+^ T cells and CD4^+^ T cells. CD4^+^ T cells were analyzed for expression of activation and adhesion/migration-related molecules, shown exemplary for CD69.

**Fig. S4.**

**Supplemental Fig. 4: Total protein concentration in murine serum samples.** The total protein concentration in experimentally used serum samples from tMCAO mice was determined via Bicinchoninic Acid assay (BCA). Analysis revealed no significant difference between total protein from IgG- and αPK-treated mice 7 days post-stroke. Data presented as mean ± SD (*n* = 4). Statistical significance was determined by unpaired Student’s *t*-test.

**Fig. S5.**

**Supplemental Fig. 5: Plasma kallikrein and Des-Arg^9^-bradykinin increase the expression of adhesion/migration-associated molecules on CD4^+^ T cells from male mice *in vitro*.** Splenic CD4^+^CD25^-^ T cells from naïve male C57BL/6 mice were stimulated with αCD3/αCD28 in the presence of 0, 0.1, 1 or 10 µg/ml (A) plasma kallikrein (PK) or (B) Des-Arg^9^-bradykinin (Des-Arg^9^-BK). CD4^+^ T cells were analyzed by flow cytometry for expression of adhesion/migration-associated molecules (48 h). Data represent two independent experiments (mean ± SD, *n* = 6). Statistical significance was determined by RM one-way ANOVA or Friedman test, * *p* < 0.05, ** *p* < 0.01.

**Fig. S6.**

**Supplemental Fig. 6: Pre-treatment of CD4^+^ T cells with Des-Arg^9^-BK does not alter the transendothelial electrical resistance of bEnd3 cells *in vitro*.** Splenic CD4^+^CD25^-^ T cells from naïve C57BL/6 mice were stimulated with αCD3/αCD28 in the absence (T cells) or in the presence of 10 µg/ml Des-Arg^9^-BK (T cells pre-Des-Arg^9^-BK) for 48 h. Then, cells were washed and 0.5 x 10^6^ cells/well were transferred into transwell inserts (8 µm pore size) with membranes pre-coated with endothelial cells (bEnd3; 0.5 x 10^5^ cells/well). Furthermore, Des-Arg^9^-BK was additionally added directly to the transwell insert (Des-Arg^9^-BK); untreated endothelial cells served as control (w/o). The lower chamber contained 100 ng/ml SDF-1β. Transendothelial electrical resistance (TEER) was measured after (A) 2.5 h and (B) 4 h. Data represent four independent experiments (mean ± SD, *n* = 4). Statistical significance was determined by RM one-way ANOVA, ** *p* < 0.01.

**Fig. S7.**

**Supplemental Fig. 7:  Treatment with plasma kallikrein-neutralizing antibody increases frequencies of activated T cells among circulating CD45^+^ leukocytes after stroke.** (A) C57BL/6 mice were subjected to 60-min transient middle cerebral artery occlusion (tMCAO). Mice were treated with isotype immunoglobulin G (IgG) or plasma kallikrein-neutralizing antibody (αPK) at days 3 and 4 (10 µg) and days 5 to 7 (5 µg). On day 7, blood was collected and the frequencies of CD4^+^ T cells co-expressing (B) activation markers and (C) adhesion/migration-associated molecules among viable CD45^+^ leukocytes were analyzed by flow cytometry. Data represent three independent experiments (mean ± SD, *n* = 10-12). Statistical significance was determined by unpaired Student’s *t*-test or Mann-Whitney test,
* *p* < 0.05, ** *p* < 0.01, *** *p* < 0.001. Created with BioRender.com

**Fig. S8.**

**Supplemental Fig. 8: Plasma kallikrein-neutralizing antibody does not affect CD4^+^ T cell responses *in vitro*.** Splenic CD4^+^CD25^-^ T cells from naïve C57BL/6 mice were stimulated with αCD3/αCD28 in the presence of 0, 10, or 100 ng/ml plasma kallikrein-neutralizing antibody (αPK). CD4^+^ T cells were analyzed by flow cytometry for (A) the expression of adhesion/migration-associated molecules (48 h) and (B) cytokine production (72 h). Data represent three independent experiments (mean ± SD, *n* = 9). Statistical significance was determined by RM one-way ANOVA or Friedman test.

**Fig. S9.**

**Supplemental Fig. 9: Frequencies of CD3^+^ T cells in spleen and cervical lymph nodes of stroke mice treated with plasma kallikrein-neutralizing antibody.** (A) C57BL/6 mice were subjected to 60-min transient middle cerebral artery occlusion (tMCAO). Mice were treated with isotype immunoglobulin G (IgG) or a plasma kallikrein-neutralizing antibody (αPK) at days 3 and 4 (10 µg) and days 5 to 7 (5 µg). (B) On day 7, immune cells from the spleen and cervical lymph nodes (LN) were isolated and analyzed by flow cytometry for frequencies of CD3^+^ T cells. Data represent two independent experiments (mean ± SD, *n* = 6-7). Statistical significance was determined by unpaired Student’s *t*-test, * *p* < 0.05. Created with BioRender.com

**Fig. S10.**

**Supplemental Fig. 10: Plasma kallikrein and Des-Arg^9^-bradykinin decrease the apoptosis of CD4^+^ T cells *in vitro*.** Splenic CD4^+^CD25^-^ T cells from naïve C57BL/6 mice were stimulated with αCD3/αCD28 in the presence of 0, 0.1, 1 or 10 µg/ml (A) plasma kallikrein (PK) or (B) Des-Arg^9^-bradykinin (Des-Arg^9^-BK). CD4^+^ T cells were analyzed by flow cytometry for late apoptosis (48 h). Data represent two independent experiments (mean ± SD, *n* = 6). Statistical significance was determined by RM one-way ANOVA or Friedman test, * *p* < 0.05, ** *p* < 0.01, *** *p* < 0.001.

**Fig. S11.**

**Supplemental Fig. 11: Delayed blocking of bradykinin signaling does not reverse the effects of Des-Arg^9^-bradykinin treatment on CD4^+^ T cells *in vitro*.** Splenic CD4^+^CD25^-^ T cells from naïve C57BL/6 mice were stimulated with αCD3/αCD28 in the presence of 1 µg/ml Des-Arg^9^-bradykinin (Des-Arg^9^-BK) for 72 h. Subsequently, cells were restimulated with αCD3/αCD28 in the presence of 20 µM Lys-(Des-Arg^9^,Leu^8^)-bradykinin (αB1R) and/or 1 µg/ml Des-Arg^9^-BK. Control CD4^+^CD25^-^ T cells were restimulated with αCD3/αCD28 alone. After restimulation, CD4^+^ T cells were analyzed by flow cytometry for (A) activation markers (24 h), (B) adhesion/migration-associated molecules (48 h), and (C) cytokine production (72 h). Data represent two independent experiments (mean ± SD, *n* = 3-6). Statistical significance was determined by paired Student’s *t*-test or Wilcoxon test, comparing Des-Arg^9^-BK treatment alone with Des-Arg^9^-BK treatment in combination with αB1R, * *p* < 0.05.

**Fig. S12.**

**Supplemental Fig. 12: The bradykinin receptor 1 antagonist Lys-(Des-Arg^9^,Leu^8^)-bradykinin attenuates Des-Arg^9^-bradykinin-induced pro-inflammatory effects on CD4^+^ T cells *in vitro*.** Splenic CD4^+^CD25^-^ T cells from naïve C57BL/6 mice were stimulated with αCD3/αCD28 in the presence of 10 µM Lys-(Des-Arg^9^,Leu^8^)-bradykinin (αB1R) and/or 1 µg/ml Des-Arg^9^-bradykinin (Des-Arg^9^-BK). Control CD4^+^CD25^-^ T cells were stimulated with αCD3/αCD28 alone. CD4^+^ T cells were characterized for adhesion/migration-associated molecules after 48 h by flow cytometry. Data represent one experiment (mean ± SD, *n* = 3). Statistical significance was determined by paired Student’s *t*-test, comparing Des-Arg^9^-BK treatment alone with Des-Arg^9^-BK treatment in combination with αB1R, or comparing the untreated control with Des-Arg^9^-BK treatment, * *p* < 0.05, ** *p* < 0.01.

**Supplemental Tables**

**Supplemental Table 1.** Patient cohort.

| **Cohort** | **2 – 4 days post-stroke** | **5 – 7 days post-stroke** | **Healthy controls** |
| --- | --- | --- | --- |
| **Age (range)** | 45-93 | 32-93 | 30-65 |
| **Age (mean ± SEM)** | 70.3 ± 14.4 | 71.4 ± 16.0 | 49.8 ± 10.3 |
| **Sex distribution (m/f)** | 6 / 5 | 7 / 6 | 6 / 6 |
| **NIHSS @ sampling (range)** | 0-16 | 0-12 | n/a |
| **NIHSS (mean ± SEM)** | 3.8 ± 4.5 | 4.4 ± 3.4 | n/a |
| **Acute treatment**  **rt-PA/rt-PA & EVT/none** | 4 / 2 / 5 | 4 / 1 / 8 | n/a |
| **Comorbidities** | Common comorbidities included AH, DM, AF, CKD, hyperlipidemia, CAD, nicotine abuse | Common comorbidities included AH, AF, DM, CKD, hyperlipidemia, CAD, ACI stenosis, PFO | No relevant comorbidities reported; occasional adiposity, nicotine abuse |
| **Premedication** | Antihypertensives, statins, antidiabetics, anticoagulants, PPIs | Antihypertensives, statins, anticoagulants, antidiabetics, CNS-active drugs | None / minimal |

**Supplemental References**

1.	Szepanowski R, Haupeltshofer S, Vonhof S, Frank B, Kleinschnitz C, Casas A, editors. Thromboinflammatory challenges in stroke pathophysiology. Seminars in Immunopathology; 2023: Springer.
